# Widespread FRA1-Dependent Control of Mesenchymal Transdifferentiation Programs in Colorectal Cancer Cells

**DOI:** 10.1371/journal.pone.0088950

**Published:** 2014-03-21

**Authors:** Jeannine Diesch, Elaine Sanij, Omer Gilan, Christopher Love, Hoanh Tran, Nicholas I. Fleming, Jason Ellul, Marcia Amalia, Izhak Haviv, Richard B. Pearson, Eugene Tulchinsky, John M. Mariadason, Oliver M. Sieber, Ross D. Hannan, Amardeep S. Dhillon

**Affiliations:** 1 Research Division, Peter MacCallum Cancer Centre, Melbourne, Victoria, Australia; 2 Department of Biochemistry and Molecular Biology, Bio21 Institute, University of Melbourne, Victoria, Australia; 3 Sir Peter MacCallum Department of Oncology, University of Melbourne, Victoria, Australia; 4 Department of Pathology, University of Melbourne, Victoria, Australia; 5 Walter and Eliza Institute of Medical Research, Victoria, Australia; 6 Ludwig Institute for Cancer Research, Victoria, Australia; 7 Faculty of Medicine, Bar-Ilan University, Tel-Aviv, Israel; 8 School of Cancer Studies and Molecular Medicine, University of Leicester, Leicester, United Kingdom; 9 Department of Biochemistry and Molecular Biology, Monash University, Victoria, Australia; 10 School of Biomedical Sciences, University of Queensland, Queensland, Australia; Technische Universität München, Germany

## Abstract

Tumor invasion and metastasis involves complex remodeling of gene expression programs governing epithelial homeostasis. Mutational activation of the RAS-ERK is a frequent occurrence in many cancers and has been shown to drive overexpression of the AP-1 family transcription factor FRA1, a potent regulator of migration and invasion in a variety of tumor cell types. However, the nature of FRA1 transcriptional targets and the molecular pathways through which they promote tumor progression remain poorly understood. We found that FRA1 was strongly expressed in tumor cells at the invasive front of human colorectal cancers (CRCs), and that its depletion suppressed mesenchymal-like features in CRC cells *in vitro*. Genome-wide analysis of FRA1 chromatin occupancy and transcriptional regulation identified epithelial-mesenchymal transition (EMT)-related genes as a major class of direct FRA1 targets in CRC cells. Expression of the pro-mesenchymal subset of these genes predicted adverse outcomes in CRC patients, and involved FRA-1-dependent regulation and cooperation with TGFβ signaling pathway. Our findings reveal an unexpectedly widespread and direct role for FRA1 in control of epithelial-mesenchymal plasticity in CRC cells, and suggest that FRA1 plays an important role in mediating cross talk between oncogenic RAS-ERK and TGFβ signaling networks during tumor progression.

## Introduction

Local invasion and metastasis of colorectal and some other carcinoma types is thought to involve transient remodeling of the epithelial tumor cell phenotype to an invasive mesenchymal-like state, characterized by defects in tight and adherens junction formation, increase in intermediate filament proteins (e.g. vimentin), and elevated expression of proteases mediating degradation of the extracellular matrix. Such epithelial-mesenchymal transitions (EMT) and mesenchymal-epithelial transitions (MET) involve extensive and highly coordinated changes in gene expression, regulated by complex signalling and transcription factor networks that engage master EMT transcription factors belonging to the basic helix-loop-helix (bHLH), Snail, Twist and ZEB families [1]–[3].

Colorectal cancer (CRC) is a genetically heterogeneous disease whose metastatic spread to the liver, lung, peritoneal cavity and bones poses major clinical problems [4]. More than 50% of CRCs harbor oncogenic mutations in the *KRAS* or *BRAF* genes, which drive persistent activation of the ERK MAPK pathway. These mutations generally arise at a relatively early stage during adenoma-carcinoma progression, following functional loss of the tumor suppressor APC, and their high incidence of concordance in primary and metastatic cancers suggests that they contribute to both tumor initiation and progression [5]–[8]. At later stages, some tumors acquire mutations (*TGFBR2*, *SMAD2*, *SMAD3* or *SMAD4*) that disrupt signaling via the TGFβ pathway, which provides growth inhibitory signals in the normal intestinal epithelium.

In CRC, EMT-like events are strongly associated with budding, a pathological phenomenon observed in 20–40% of cases, in which tumor cells detach from the invasive front and invade into the surrounding basement membrane [9], [10]. The interplay between EMT and MET has also been suggested to underlie the observation that basement membrane expression is often lost at the invasive front of CRCs, but regained in metastases [11]. More recently, several studies have identified EMT-related gene expression signatures as a common occurrence in primary CRCs, which are strongly associated with poor prognosis and resistance to targeted therapies [12]–[15].

One of the major pathways involved in induction and maintenance of EMT events during development and tumorigenesis is the TGFβ pathway. Although activation of the pathway is growth inhibitory in normal epithelial cells, oncogenic RAS-ERK signaling has been reported to induce a paradoxical switch in its role from tumour suppressor to pro-metastatic factor, in part through activation of EMT-related programs [2], [16]–[19]. The mechanisms underlying pro-malignant cross talk between the RAS and TGFβ pathways during CRC progression are not well understood. Perturbation of the TGFβ pathway is a frequent event in CRC. Complete inactivation of TGFβ signaling through mutation of the type II TGFβ receptor (*TGFBR2*) locus is a common occurrence in the 15% of CRCs displaying DNA microsatellite instability (MSI), which ironically are associated with favorable prognostic outcomes [20]. The remaining 85% of microsatellite stable (MSS) cancers display chromosomal instability (CIN), and often (∼50% of cases) harbor mutations in the *SMAD4* gene, and less frequently the *SMAD2* and *SMAD3* genes [21]. Recent studies have shown that the *TGFBR2* genotype is the major factor determining a lack of EMT-like responses to TGFβ1 in MSI-positive tumors, whereas CRC cells with *SMAD4* mutations retain the ability to undergo EMT upon TGFβ1 treatment through coupling with the ERK pathway [22]. The nature of ERK pathway effectors required for these responses are presently unclear.

The Activator Protein-1 complex is a key regulator of transcriptional responses induced by various cancer-associated signalling pathways. It consists of homo- or hetero-dimers of Fos, Jun, ATF and MAF family members, whose activities are strongly influenced by oncogenic signaling events. Activating mutations in RAS pathway components have been shown to induce overexpression of the Fos family member FRA1, a highly unstable protein that is expressed at low levels in normal cells [23], [24]. Transcriptional induction and post-translational stabilization of FRA1 by oncogenic RAS-ERK signaling increases the relative abundance of FRA1 containing AP-1 dimers, which has been causally linked enhanced migration and invasion of CRC and multiple other carcinoma cell types, including breast, lung, bladder, head and neck, thyroid and brain [6], [23]–[34]. In addition to the RAS-ERK pathway, FRA1 expression is induced by the Wnt/β-catenin pathway in CRC cells [40], [41], suggesting that it may play a role in integrating signaling through these pathways during CRC progression.

In the present study, we applied a genome-wide approach to better understand the nature of transcriptional programs underlying the pro-malignant actions of FRA1 in CRC. We found that FRA1 binds and regulates expression of a clinically relevant cohort of genes associated with EMT in invasive CRC cells, with stable FRA1 knockdown invoking a MET-like phenotypic switch. FRA1 was strongly enriched in budding tumor cells at the invasive front of human CRCs, and expression of its pro-mesenchymal targets identified a subset of primary cancers with poor prognosis. Mechanistically, we found that activation of mesenchymal genes involved FRA1-dependent regulation and cooperation with TGFβ signaling networks. Collectively, our findings suggest that FRA1 plays a widespread and direct role in transcriptional control of epithelial-mesenchymal programming during CRC progression.

## Results

### FRA1 is enriched in tumor cells at the invasive front of human CRCs

**Figure 1:**
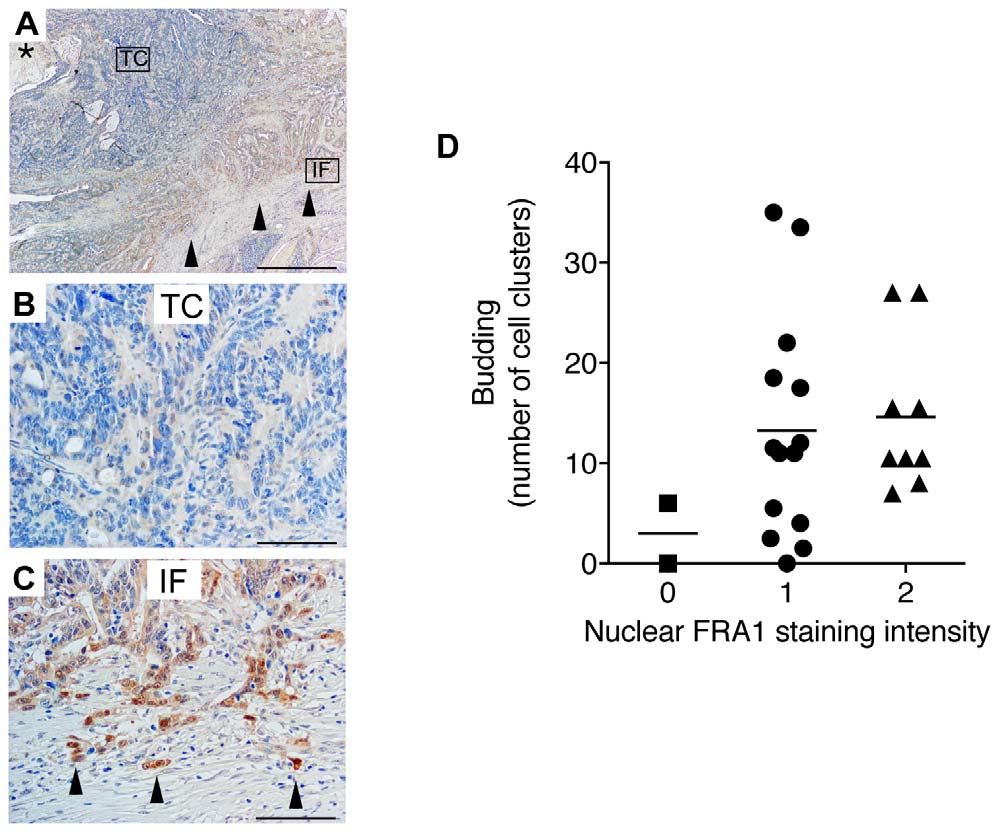
Enrichment of FRA1 in tumor cells at the invasive front of human CRCs. (A) Low power image of a representative colorectal carcinoma stained with an antibody detecting FRA1. The asterisk indicates the lumen, while the arrowheads indicate the deep invasive front. Scale bar represents 1 mm. (B and C) High power images of the tumor centre (TC) and invasive front (IF) shown in (A). Arrowheads indicate tumor buds. Scale bar represents 10 µM. (D) Relationship between the intensity of nuclear FRA1 expression and the tumor budding marker, cytokeratin AE1/AE3 in 25 CRC cases.

Although it was previously reported that FRA1 is more highly expressed in CRCs than the normal colorectal epithelium [35], its relationship with tumor pathology has not been established. Using immunohistochemistry, we detected FRA1 immunoreactivity in 20 out of 25 primary tumor specimens. In contrast to its weak expression in the center of tumors, cells at the invasive front exhibited strong FRA1 staining (Figure 1A–1C), including cytokeratin AE1/AE3 positive clusters of cells [36] that had detached from the tumor bulk (Figure 1D). This latter feature is indicative of tumor budding, a phenomenon associated with the acquisition of mesenchymal-like features by CRC cells, and is an independent predictor of lymph node metastasis, vascular and lymphatic invasion, distant metastasis, local recurrence and poor disease-free survival [9].

### FRA1 regulates mesenchymal-like features in CRC cells

**Figure 2:**
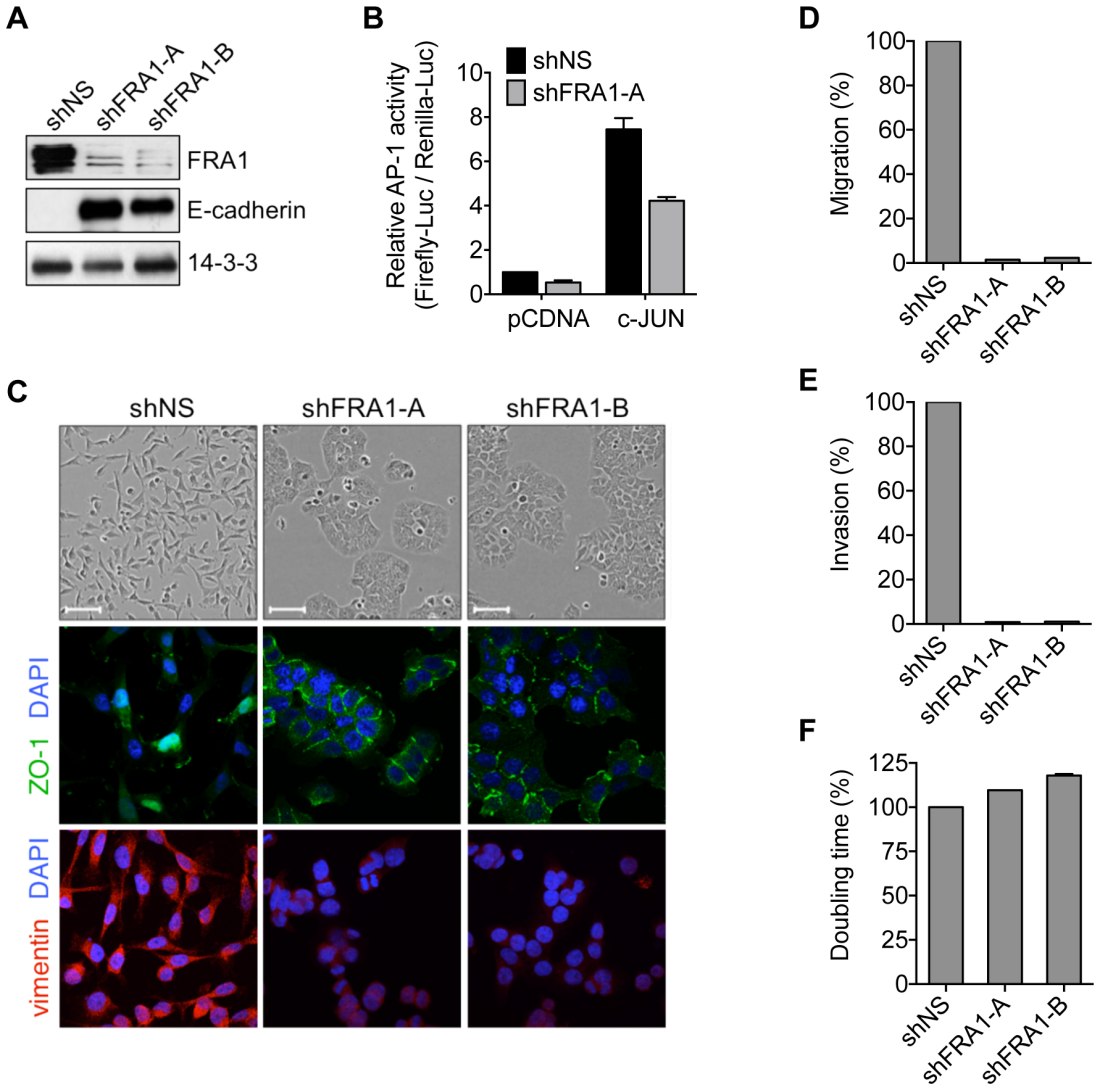
FRA1 knockdown suppresses mesenchymal-like features in CRC cells. (A) Immunoblot analysis of FRA1 and E-cadherin levels in BE cells stably transduced with a non-silencing control (shNS) or FRA1-targeting shRNAs (shFRA1-A and -B). (B) Effect of FRA1 knockdown on basal and c-Jun-induced AP-1 reporter gene activity in BE cells. (C) Phase-contrast images (top row) and immunofluorescence staining for DAPI with ZO-1 (middle row) or vimentin (bottom row) on the cells from (A). Scale bar represents 10 µM. (D–F) Analysis of *in vitro* migration, invasion and proliferation in cells from (A). Error bars represent S.E.M. for 3 independent experiments.

Previous investigations on the pro-invasive actions of FRA1 in CRC have used the BE cell line model, which comprises highly invasive mesenchymal-like cells that harbor *KRAS*/*BRAF* mutations driving high endogenous FRA1 expression [33], [37]. Consistent with its role as a major contributor to AP-1 activity in these cells, stable knockdown of FRA1 using 2 distinct shRNAs constructs reduced both basal and c-Jun stimulated AP-1 reporter gene activation (Figure 2A and 2B). Phenotypically, FRA1 depletion invoked a striking mesenchymal to epithelial-like morphological switch, with cells acquiring a flattened appearance, regaining expression of the epithelial differentiation marker E-cadherin, and forming tight junctions staining positively for ZO-1 (Figure 2A and 2C). In addition, FRA1-depleted cells almost completely lost their capacity to migrate and invade *in vitro*, but their proliferation rates remained unchanged (Figure 2D–2F). Together, these findings suggest a requirement for FRA1 to maintain CRC cells in a mesenchymal-like state.

### EMT-related genes are a major class of direct FRA1 targets in CRC cells

**Figure 3:**
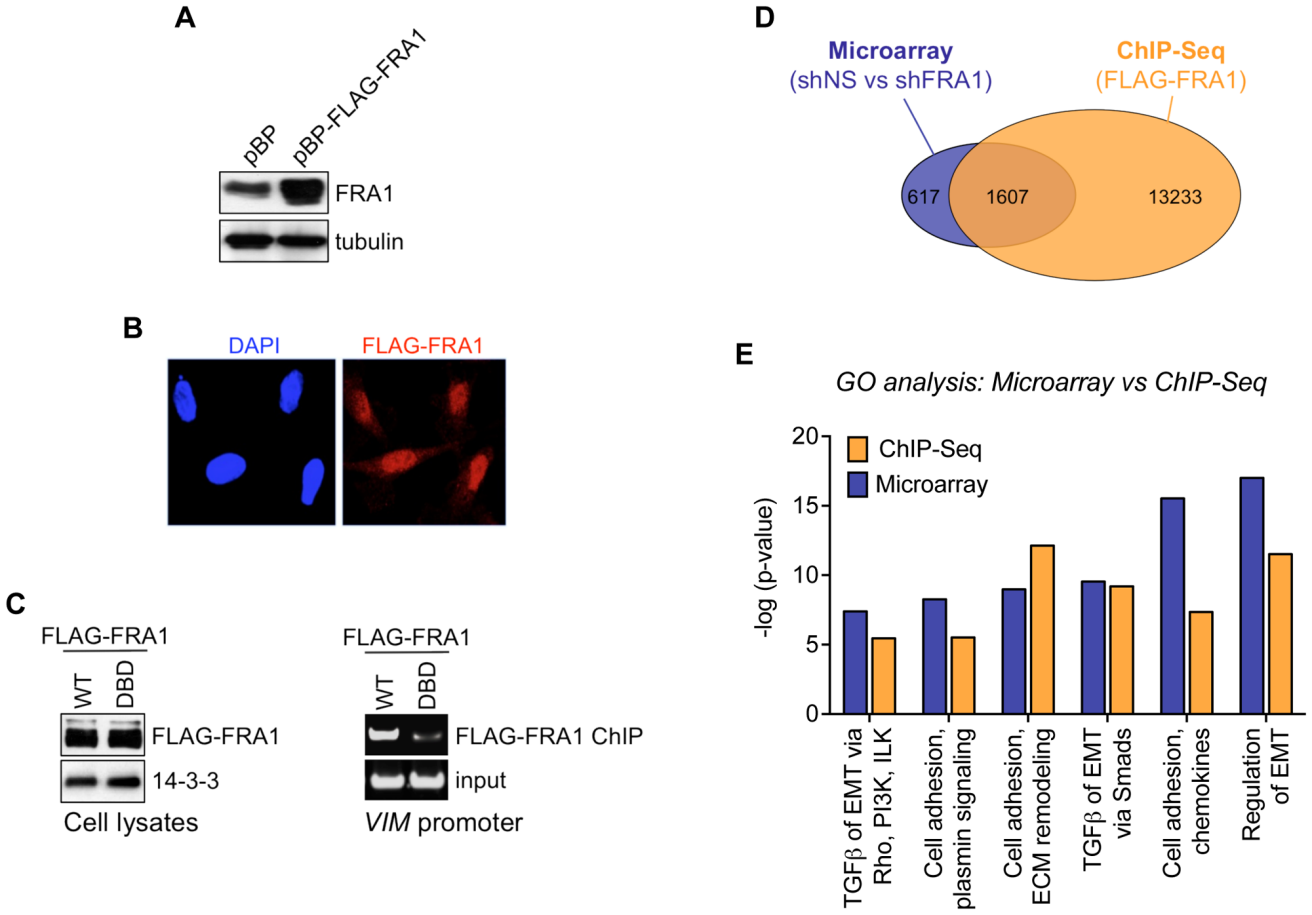
Genome-wide analysis of direct FRA1 transcriptional targets in CRC cells. (A) Immunoblot analysis of FRA1 expression in BE cells stably transduced with a FLAG-FRA1 expression construct of vector (pBP) control. (B) Immunofluorescence analysis of FLAG-FRA1 localization in cells from (A). (C) ChIP analysis comparing binding of wild-type and DNA binding defective (DBD) FLAG-FRA1 proteins to the *VIM* promoter in BE cells. (D) Intersection of data from microarray (FRA1 shRNA) and ChIP-Seq (FLAG-FRA1) analysis in BE cells. The ChIP-Seq results represents genes at which FRA1 binding was enriched >5-fold relative to input control within 5 kb of the transcription start site (TSS). Only annotated genes undergoing at least a 2-fold change in expression upon FRA1 silencing (p<0.05) were considered, and genes associated with multiple FLAG-FRA1 peaks were only counted once. (E) Ontological analysis of genes identified after intersection of the microarray and ChIP-Seq datasets. Genes were clustered into major biological pathways using GeneGo.

To identify direct transcriptional targets and molecular pathways through which FRA1 controls mesenchymal-like features in tumor cells, we performed genome-wide ChIP-Seq and transcriptome analysis to search for genes at which FRA1 binding was enriched, and whose expression was regulated by FRA1 in BE cells. Despite several attempts to optimize the ChIP assay using antibodies targeting endogenous FRA1, we were unable to recover sufficient chromatin to perform subsequent high-throughput sequencing. We therefore generated BE cell lines moderately (∼5-fold higher than endogenous FRA1) overexpressing a FLAG-FRA1 protein, which was localized in the nucleus (Figure 3A,B). In contrast to a DNA binding defective variant, wild-type FLAG-FRA1 demonstrated strong enrichment at the promoter of *VIM*, a previously identified direct FRA1 target (Figure 3C) [38].

Using microarrays, we found that FRA1 knockdown in BE cells significantly up-regulated expression of 1392 genes while reducing expression of 832 genes by at least 2-fold (Figure 3D). ChIP-Seq analysis revealed that 72% percent of the FRA1-regulated genes contained loci significantly enriched for FRA1 binding (>5-fold) within 5 kb of their transcription start site (TSS). To identify major functional classes of FRA1 target genes, we intersected the microarray and ChIP-Seq data and interrogated overlapping genes for enrichment of gene ontology terms (using GeneGo). Of the top 6 groups identified using this approach, 3 were significantly enriched for genes associated with EMT-related processes, while a further 3 groups featured adhesion-related genes (Figure 3E). These findings indicated that EMT and adhesion are two major processes under direct FRA1 transcriptional control in CRC cells.

**Figure 4:**
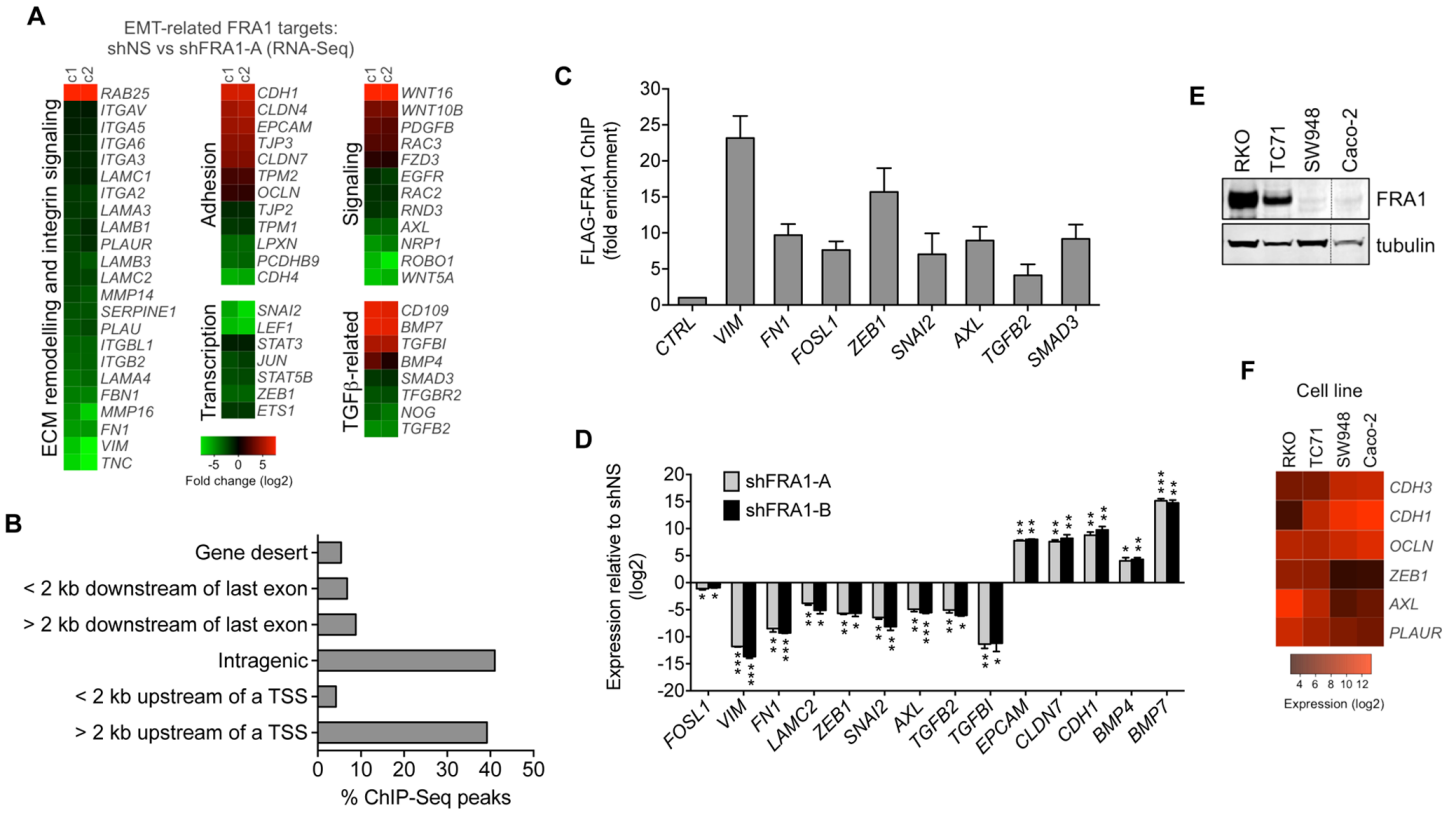
Characterization of EMT-related FRA1 transcriptional targets. (A) Heat map showing different functional groups of EMT-related genes bound and regulated by FRA1 (FRA1^EMT^ genes). Data from RNA-Seq analysis of two clones of BE shFRA1-A cells (n = 4 for each cell line) was normalised relative to shControl cells. Regions shown in red represent genes associated with an epithelial state that were upregulated upon FRA1 silencing (log fold-change<−1, p<0.05), while green regions represent mesenchymal-type genes repressed by FRA1 silencing (log fold-change>1, p<0.05). (B) Distribution of genomic FLAG-FRA1 binding sites identified by ChIP-Seq relative to a corresponding gene. The number of reads identified for each region is expressed as a percentage. (C) ChIP-qPCR analysis of FLAG-FRA1 binding to genomic regions in selected FRA1^EMT^ genes. Data represent relative enrichment compared to parental BE cells. A region of the miRNA-21 gene not bound by FLAG-FRA1 was used as negative control (*CTRL*). (D) qRT-PCR analysis of selected FRA1^EMT^ genes in BE cells stably transduced with one of two independent shRNAs targeting FRA1. Data are represented relative to expression levels in cells shNS cells. Student's t-test was used for all comparisons (^*^p<0.05, ^**^p<0.01, ^***^p<0.001). Error bars represent S.E.M. for 3 independent experiments. (E) FRA1 protein levels and (F) expression of epithelial and mesenchymal marker genes in a panel of CRC cell lines.

Based on the phenotypic changes resulting from FRA1 knockdown in BE cells, and the association between tumour budding and EMT in CRC [9], we chose to focus on the involvement of FRA1 in regulating EMT events in CRC. Collectively the EMT-related genes bound and regulated by FRA1 (herein termed FRA1^EMT^ genes) encoded a diverse array of proteins involved cell adhesion, signal transduction, transcription, cytoskeletal and extracellular matrix remodeling, as well as components of TGFβ signaling networks (Figure 4A and Table S1 in File S1). These genes broadly comprised a pro-mesenchymal subset whose expression was promoted by FRA1 and an epithelial subset that was repressed by FRA1. Many of the genes contained multiple loci occupied by FRA1, which were primarily located within intronic regions of the gene body and in distal upstream sites (Figure 4B and Table S2 in File S1). Motif analysis revealed that 58% of the genes contained at least one FRA1 binding site significantly enriched for a consensus AP-1 binding sequence (p<0.0001, Table S3 in File S1). We also noted significant enrichment (p<0.001) for putative MEF-2 motifs in the EMT-related targets identified in the ChIP-Seq analysis. Finally, we performed ChIP and qRT-PCR analysis to confirm enrichment of FRA1 at several loci identified by ChIP-Seq (Figure 4C) and FRA1-dependent regulation of selected targets in BE cells (Figure 4D).

### Expression of pro-mesenchymal FRA1 targets predicts poor clinical outcomes

**Figure 5:**
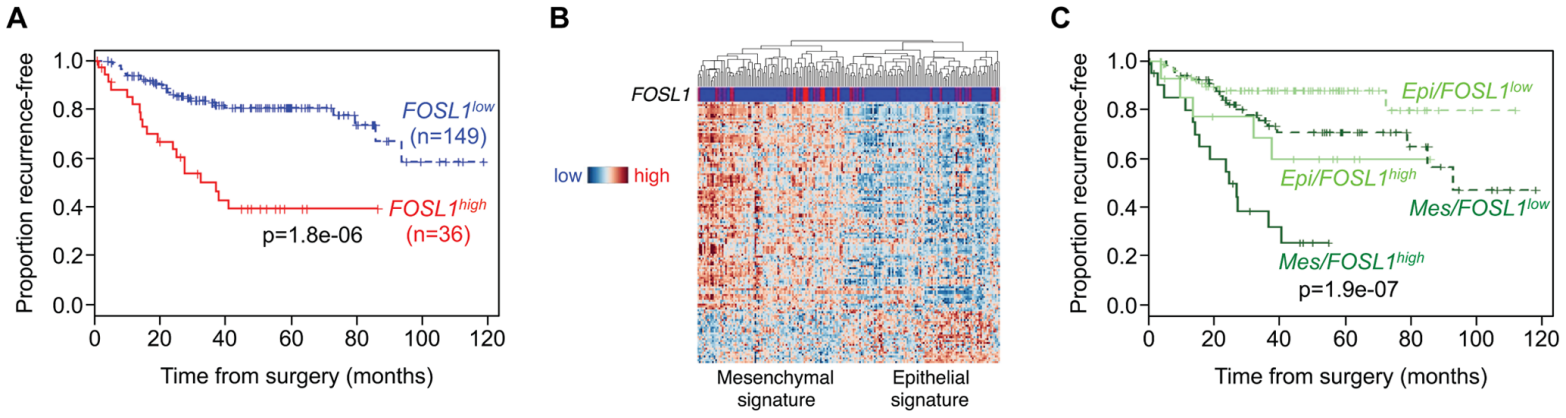
Clinical significance of FRA1 and FRA1^EMT^ genes in CRC. (A) Kaplan-Meier plots of recurrence-free survival in stage B and C CRC patients according to expression of the FRA1 gene (*FOSL1*). (B) Unsupervised clustering of stage B and stage C CRCs based on FRA1^EMT^ genes encompassing concordant probesets exhibiting significant expression differences between the two main groups. Clustering divides cancers into groups with mesenchymal and epithelial profiles. Samples are arranged along the X-axis and genes along the Y-axis. Genes are grouped into those downregulated (blue) or upregulated (orange) upon FRA1 knockdown in BE cells relative to the mean- and sample-centered scaled expression. (C) Kaplan-Meier plots of recurrence-free survival in stage B and C CRC patients based on expression of both *FOSL1* (low vs high) and mesenchymal (Mes, dark green) or epithelial (Epi, light green) subsets of FRA1^EMT^ genes. The log-rank test was used for comparisons.

To assess the potential clinical relevance of the EMT-related FRA1 targets, we examined the relationship between their expression and CRC prognosis by interrogating existing microarray data from 185 stage B and C cases [12]. Unsupervised clustering of the data revealed that tumors could be classified into epithelial- and mesenchymal-like subgroups displaying gene expression differences highly concordant with the FRA1^EMT^ signature (Figure 5B and Table S4 in File S1), with 77% of genes (137 probesets) showing directional changes consistent with those identified upon FRA1 knockdown in BE cells.

We also found that high levels of FRA1 gene (*FOSL1*) expression independently predicted poor recurrence-free survival and was associated with a higher T-stage, an index of advanced tumor invasion (Figure 5A and Table S5 in File S1). Although *FOSL1* expression was detected in both epithelial- and mesenchymal-like tumors, its expression was significantly higher (p<0.05) in the latter group. Expression of pro-mesenchymal FRA1 targets was enriched in 48.6% (90/185) of primary tumours, which had an earlier diagnosis age (median 64 vs 70 years, p = 0.005) and higher lymph node stage (N2 76% vs 24%, p = 0.0098) compared to epithelial-type tumors (Table S6 in File S1).

Integrating data on *FOSL1* expression and the FRA1^EMT^ signature significantly improved prediction of recurrence risk, broadly separating patients into 3 outcome-based groups (Figure 5C and Table S7 in File S1): (i) A good prognosis group consisting of *FOSL1* ^low^ epithelial-type cancers, (ii) an intermediate prognosis group comprising *FOSL1* ^low^ mesenchymal-type cancers and *FOSL1* ^high^ epithelial-type cancers, and (iii) a poor prognosis group of *FOSL1* ^high^ mesenchymal-type cancers. These findings suggest that the combination of elevated *FOSL1* and pro-mesenchymal FRA1 target gene expression in primary tumors provides a robust predictor of adverse outcomes in CRC patients.

### Cross talk between FRA1 and TGFβ signalling controls mesenchymal gene expression

**Figure 6:**
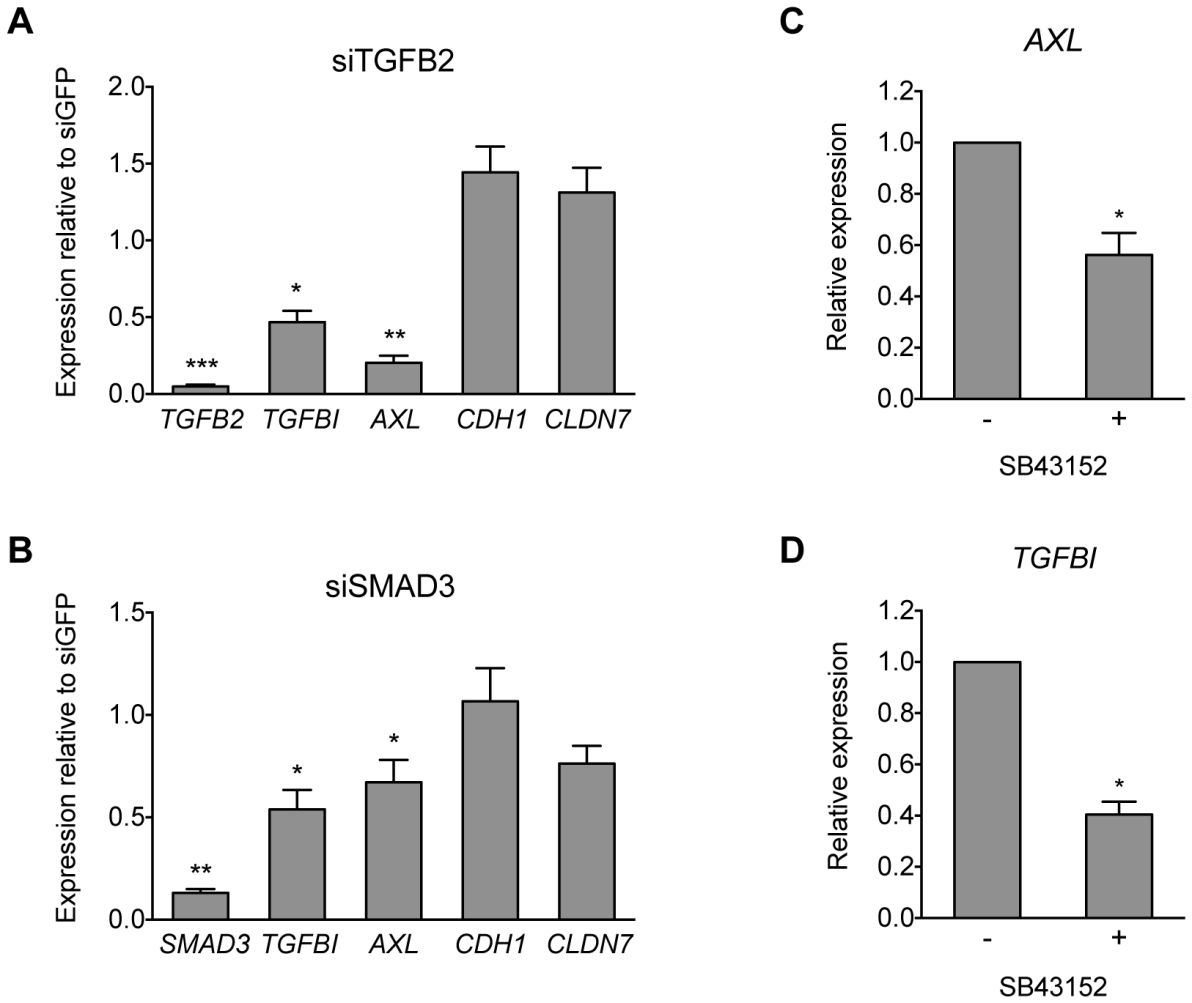
A FRA1-dependent autocrine TGFβ2 loop promotes mesenchymal gene expression in BE CRC cells. (A and B) Expression of selected mesenchymal (*TGFBI*, *AXL*) and epithelial (*CDH1*, *CLDN7*) FRA1^EMT^ genes upon transient knockdown of the TGFβ pathway FRA1 targets *TGFB2* and *SMAD3* using siRNA pools in BE cells. Data are represented relative to levels of these genes in cells transfected with siRNAs targeting GFP. (C) Effects of the TGFβ receptor inhibitor SB43152 (10 µM for 72 h) on expression of a selected mesenchymal FRA1^EMT^ (*TGFBI*, *AXL*) genes in BE cells. Student's t-test was used for all comparisons (^*^p<0.05, ^**^p<0.01, ^***^p<0.001). Error bars represent S.E.M. for 3 independent experiments.

Given their association with adverse clinical outcomes (Figure 5), we next sought to gain a deeper mechanistic insight into FRA1-dependent control of mesenchymal expression programs in CRC cells. In particular, we wondered if in addition to directly binding and regulating their transcription, FRA1 could promote expression of pro-mesenchymal genes by modulating the activities of EMT-associated signaling pathways whose components we had identified as direct FRA1 targets. The most highly represented of these were genes acting in TGFβ signaling networks, including those encoding activating (*TGFB2*) and inhibitory ligands (*BMP4*, *BMP7*), whose expression was promoted or repressed by FRA1, respectively (Figure 4A, 4C and 4E). Being the only activating ligand identified as a FRA1 target, we chose to investigate the potential contribution of TGFβ2 signaling in regulating expression of a selection of FRA1^EMT^ targets. Transient knockdown of *TGFB2* in parental BE cells significantly reduced expression of several pro-mesenchymal (*AXL*, *VIM*) but not epithelial (*CDH1*, *CLDN7*) FRA1 target genes (Figure 6A). Similar effects were also observed upon transient knockdown of another FRA1 bound target acting in the pathway, encoding the transcription factor SMAD3 (Figure 6B). To directly examine the possibility that a FRA1-dependent autocrine TGFβ2 loop was operating in these cells, they were treated with the type 1 TGFβ receptor inhibitor SB43152 to block transduction of TGFβ2 signals. Consistent with the effects of *TGFB2* knockdown, we found that expression of several pro-mesenchymal FRA1 targets (*AXL*, *VIM*, *TGFBI*) was significantly reduced after 3 days of SB43152 treatment.

**Figure 7:**
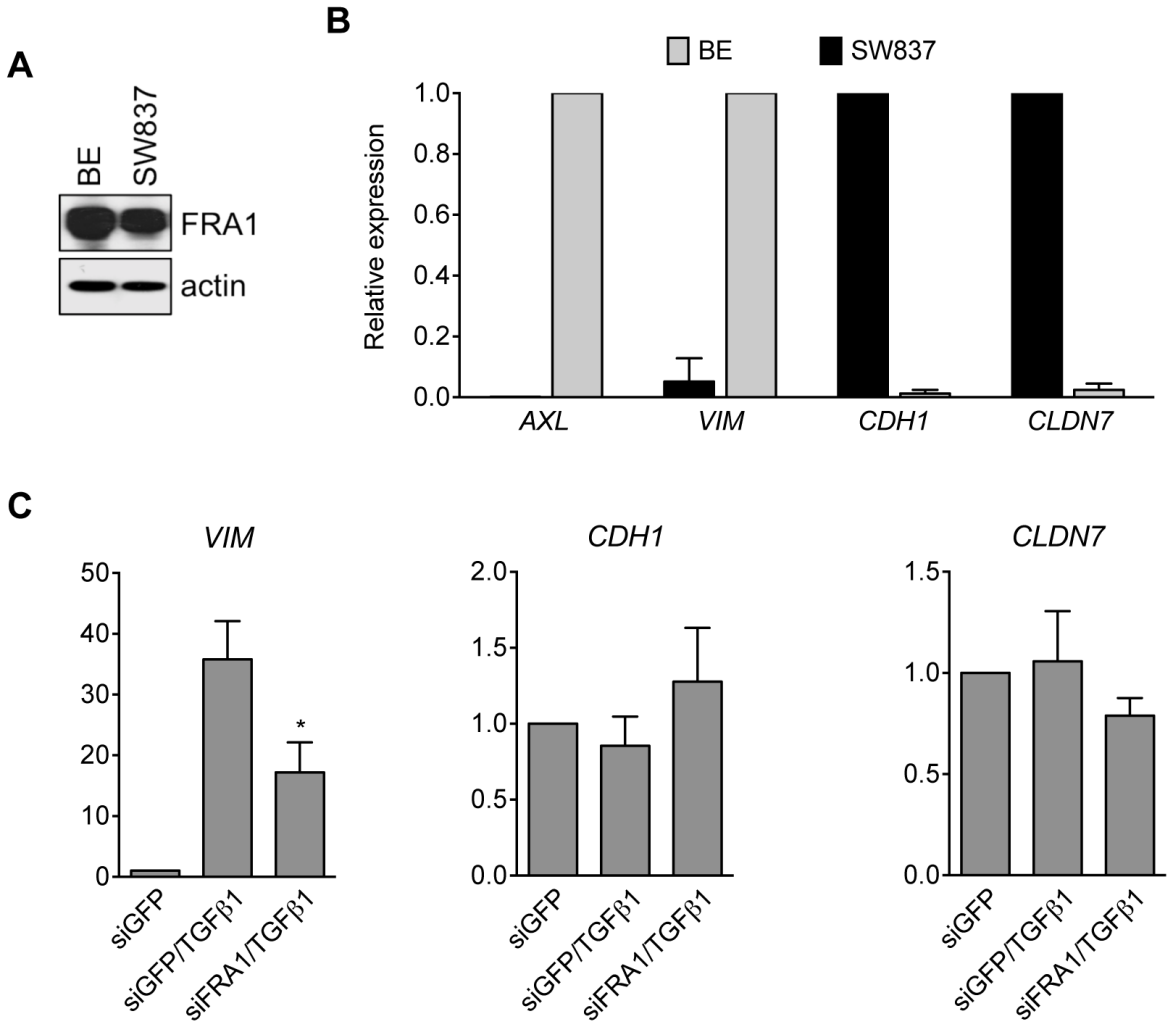
FRA1 controls pro-mesenchymal transcriptional responses induced by TGFβ in CRC cells. (A) Immunoblot analysis of endogenous FRA1 expression in BE and SW837 CRC cells. (B) qRT-PCR analysis comparing relative expression levels of a selection of mesenchymal (*AXL*, *VIM*) and epithelial (*CDH1*, *CLDN7*) FRA1^EMT^ genes in BE and SW837 CRC cells. (C) Effects of transient FRA1 knockdown on TGFβ1-induced (10 ng/mL 48 h) expression of *VIM*, *CDH1* and *CLDN7* in SW837 cells. Student's t-test was used for all comparisons (^*^p<0.05). Error bars represent S.E.M. for 3 independent experiments.

To further investigate the extent of cross talk between FRA1 and the TGFβ pathway, we assessed the expression of several mesenchymal and epithelial FRA1 targets in another *KRAS* mutant TGFβ-responsive CRC cell line, SW837. Despite FRA1 levels being elevated in these cells (Figure 7A), their expression of the pro-mesenchymal genes *VIM* and *AXL* was low when compared to BE cells, while the epithelial genes *CDH1* and *CLDN7* were highly expressed (Figure 7B). Treatment with the ligand TGFβ1 robustly induced expression of *VIM*, a response that was significantly impaired upon prior FRA1 knockdown (Figure 7C). By contrast, expression of the epithelial FRA1 targets *CDH1* and *CLDN7* was unaffected under the same conditions. These results suggest that FRA1 expression modulates the extent to which TGFβ signaling can induce pro-mesenchymal transcriptional responses in CRC cells.

## Discussion

The local invasion and metastatic spread of cancers involves specific, highly coordinated and dynamic remodeling of tumor cell gene expression through bidirectional cross talk between signaling and transcriptional networks. Much remains to be understood about how these networks are regulated by tumor-associated genetic and epigenetic lesions, and the mechanisms through which they are coordinated to induce specific changes in gene expression during invasion.

The transcription factor AP-1 has long been implicated as a central regulator of tumor cell invasion [39]. FRA1 is one of the most frequently overexpressed AP-1 proteins in solid cancers, and its ability to promote migratory and invasive traits in a variety of different tumor cell types [26]–[32] suggests that its actions involve engagement of common targets and pathways. The identity of these pathways is presently unclear, while only a handful of its direct transcriptional targets in carcinoma cells identified to date. Through analysis of its genome-wide chromatin occupancy and target gene regulation, the present study identifies genes and pathways involved in cell adhesion and EMT as major classes of direct FRA1 targets associated with CRC progression.

EMT-like gene expression signatures have been reported to correlate with poor prognosis and resistance to targeted therapies in CRC patients, but the mechanisms governing their genesis are poorly understood [12]–[15]. Our findings suggest that FRA1-regulated transcriptional events play an important role in this process, with elevated levels of pro-mesenchymal FRA1 targets associated with adverse outcomes in about half of all stage B and stage C cancers, while high levels of FRA1 gene (*FOSL1*) expression in these mesenchymal-type identifying cancers with poorest prognosis.

Pathological EMT in CRC strongly linked with tumor budding, an independent prognostic indicator of higher lymph node metastasis, vascular and lymphatic invasion, distant metastasis, local recurrence and poor disease-free survival [9]. While FRA1 expression appears relatively homogenous in tumor cell lines, we found that it was highly enriched at invasive regions and in budding cells but not the center of primary tumors. The mechanism underlying this restricted expression in tumors is presently unclear, however a similar localization pattern has been reported for the Wnt pathway transcriptional effector, β-catenin [40], [41]. As β-catenin induces transcription of the FRA1 gene in CRC cells, FRA1/β-catenin cooperativity may play an important role in controlling localized transcription of pro-invasive genes in colorectal tumors, a notion supported by our finding that FRA1 directly binds and regulates several pro-invasive β-catenin targets, including *MMP14*, *LAMC2*, *VIM* and *ZEB1* [11], [42]–[46].

The induction of EMT involves remodeling of multiple cellular processes, including adhesion, signaling, transcription, and extracellular matrix remodeling. There is growing evidence that tumor cells often exhibit only some of these changes, for example expressing a subset of mesenchymal markers while retaining epithelial features [3]. The ability of tumor cells to transit from an epithelial to mesenchymal-like state is thus likely to be highly context-specific, and require cooperativity between multiple signaling and transcriptional networks. The ability of FRA1 to bind and regulate genes involved in different EMT-associated processes suggests that it may play an important role in coordinated EMT events. Interestingly, in BE CRC cells, FRA1 binding was involved both in maintaining expression of pro-mesenchymal genes, while repressing an epithelial subset. Several potential mechanisms may contribute to these opposing effects of FRA1, including its assembly into distinct FRA1/Jun complexes within the same cells, its coupling with different signaling networks, and ability to promote expression of the master EMT transcriptional factors, ZEB1 and/or SNAI2. Additionally, FRA1 binding may result in localized changes in chromatin dynamics, a role recently ascribed to AP-1 in regulating the inducibility of glucorticoid receptor targets [47]. FRA1 may thus contribute to the generation of permissive chromatin contexts, necessary for both the reprogramming of CRC cells to a mesenchymal state, and subsequently to sustain the operation of mesenchymal programs when tumour cells disseminate. Interestingly, the major regions of AP-1 binding identified previously near glucocorticoid receptor targets and in the present study occurred upstream of target gene promoters or within introns, implicating a role for FRA1 in transcriptional control at an enhancer level and/or downstream of transcription initiation (e.g. elongation).

While stable FRA1 knockdown invoked a MET-like phenotypic change in BE cells, we have noted that many FRA1-overexpressing CRC cell lines retain epithelial features (ASD, unpublished). Thus FRA1 expression alone is not sufficient to drive EMT-like cellular changes, but may do so in cooperation with other pathways. Indeed, we identified components of several signaling networks as direct FRA1 targets in BE cells, with the most highly represented acting in the TGFβ pathway. We found that an underlying function of FRA1 in CRC cells was to positively regulate TGFβ signaling, which it could do via several mechanisms; in the mesenchymal-like BE cell line, its binding modulated expression of multiple TGFβ pathway components, including maintaining operation of an autocrine TGFβ2 loop that promoted expression of mesenchymal genes. However, in epithelial-like SW837 CRC cells where autocrine TGFβ signaling was not established, FRA1 was required for TGFβ-induced mesenchymal gene expression responses. Collectively our findings suggest that FRA1 may play an important role in coupling oncogenic RAS-ERK signaling with the TGFβ pathway to control EMT-like responses in CRC cells. We suggest that FRA1 may have a similar function in other cancers where it is overexpressed such as breast, where FRA1 has recently been shown to play an important role in regulating EMT events and metastasis [25], [48], and in which the TGFβ and RAS-ERK pathways have been reported to cooperate during EMT induction [19].

From a clinical perspective, our findings are consistent with recent work showing that induction of EMT is impaired in microsatellite instable (MSI) colon cancer cells due to the presence of *TGFBR2* mutations [22]. Interestingly, CRC cells harboring *SMAD4* mutations were found to retain the ability to undergo EMT-like changes in response to TGFβ by coupling with the ERK pathway. We suggest that FRA1 may be a critical ERK pathway effector regulating EMT-like changes in these cells. Consistent with this notion, we have found that TGFβ-mediated induction of SNAI2 in *SMAD4* mutant SW480 CRC cells is regulated FRA1 (ASD, unpublished).

In summary, the present work reveals an unexpectedly widespread and direct role for FRA1 in transcriptional control of clinically relevant programs governing epithelial-mesenchymal plasticity. We also show that these actions of FRA1 are intricately linked with its coupling to the TGFβ signaling network. Approaches to inactivate FRA1 and/or pathways through which its pro-malignant actions are mediated may hence provide an approach to modulate EMT-MET balances and impede the spread of CRC and other cancers in which the RAS-ERK pathway is hyperactive.

## Materials and Methods

### Cell culture

The BE [33], [37] and SW837 CRC cell lines were maintained in Dulbecco's modified Eagle's medium (DMEM) supplemented with 2 mM L-glutamine and 10% fetal bovine serum (FBS). Clonal cell lines stably expressing shRNAs or wild-type FLAG-FRA1 or a DNA binding defective (R112V/R123V) mutant were generated using standard retroviral transduction procedures followed by 2 weeks of puromycin selection. Recombinant human TGFβ1 and the ALK inhibitor SB43152 were from Peprotech (New Jersey, U.S.A.).

### Plasmids and antibodies

Two shRNAmirs targeting FRA1 (shFRA1 A: 5′ CCTGGTGCCAAGCATCAACA 3′ and shFRA1 B: 5′ TGGACAGTATCCCACATCCAAC 3′) were designed using the RNAi Codex database [49] and cloned into the LMP retroviral vector (Open Biosystems). The LMP vector containing a non-silencing shRNA (shControl) was a gift from Dr Gretchen Poortinga. The pBABE-puro-FLAG-FRA1 construct was generated by PCR-mediated fusion of a FLAG epitope to the N-terminus of FRA1. The following antibodies were used in this study: anti-FRA1 (R-20; Santa Cruz Biotechnology), anti-FLAG M2 (Sigma-Aldrich), anti-14-3-3 (Santa Cruz Biotechnology), anti-vimentin (Cell Signalling Technology), anti-E-Cadherin (BD Transduction Laboratories), anti-ZO-1 (BD Transduction Laboratories), anti-14-3-3 (BD Transduction Laboratories) and anti-β-catenin (BD Transduction Laboratories).

### RNA interference

The following siRNAs used in this study were purchased from Dharmacon (Melbourne, Victoria, Australia): FOSL1 ON-TARGETplus SMARTpool (L-004341-00), siFRA1 custom (5′ CACCAUGAGUGGCAGUCAG 3′), GFP Duplex I (P-002048-01), SMAD3 siGENOME SMARTpool (M-020067-00), LEF1 siGENOME SMARTpool (M-015396-00), WNT5A siGENOME SMARTpool (M-003939-01), TGFB2 siGENOME SMARTpool (M-010544-00). Cells were transfected with siRNAs at a final concentration of 25 nM using the DharmaFECT 1 reagent (Dharmacon).

### Immunohistochemistry and immunofluorescence microscopy

Immunohistochemical staining for FRA1 in tumors was performed using a rabbit polyclonal antibody (Santa Cruz sc-605, 1∶2000 dilution) and visualised using horseradish peroxidase conjugated secondary antibody and DAB substrate (Vector Laboratories). FRA1 expression was intensity was scored 0, 1 or 2, with 0 representing no detectable staining and 2 representing the strongest staining observed in the sample set. A sample of human squamous cervix epithelium was used as a positive control. IHC for epithelial cytokeratins was performed using an AE1/AE3 antibody mix (Chemicon) at a 1∶200 dilution and was visualised with horseradish peroxidase conjugated secondary antibody and DAB substrate (Vector Laboratories). Tumor budding was defined as the mean number of clusters of tumor cells (containing at least 4 cells each) adjacent to the tumor front and counted in two consecutive 40× power microscopy fields, within the region of the slide displaying most budding. FRA1 expression and the extent of tumor budding were both scored in a blinded fashion by two medical pathologists. For immunofluorescence analysis, cells were cultured on glass coverslips for 24 h prior to fixation (4% paraformaldehyde in PBS), permeabilization (0.2% Triton-X100 in PBS) and blocking (10% FBS in PBS), each for 20 min at room temperature. The cells were stained with primary antibodies (1∶200 anti-ZO-1 or 1∶75 anti-vimentin; diluted in PBS/0.1% BSA) followed by secondary antibodies (anti-mouse IgG or anti-rabbit IgG coupled to Alexa-488 or Alexa-594, Invitrogen), each for 1 h at room temperature. Nuclei were stained with DAPI (Invitrogen) prior to mounting the coverlips using Mowiol (10% Hopval 5–88, 25% glycerol, 0.1M Tris pH 8.5). Images were taken on an Olympus Fluoview FV1000 confocal microscope.

### Migration, invasion and proliferation assays

Seventy-five thousand cells were seeded in triplicate into 24-well cell culture inserts (8 µm pore, BD Biosciences) for migration assays or into BD BioCoat invasion chambers (BD Biosciences) for invasion assays. As chemoattractant, 10% FBS was added in the bottom chamber. After 24 hours, cells on the upper filter surface were removed with a cotton swab, while those on the lower surface were fixed and stained using the Diff-Quick staining kit (Lab Aids). Cell migration or invasion was quantified by counting eight random fields per filter using a light microscope (Olympus BX51). To assess proliferation, 35000 cells were seeded in quadruplicate into 24-well plates and assayed for cell density at 4 hour intervals over 72 hours using the IncuCyte™FLR live-cell imaging system (Essen BioScience). The data was analysed using the IncuCyte™ cell proliferation assay algorithm.

### Microarrays, RNA-Seq and RT-qPCR

RNA was purified from cells using the Isolate RNA Mini kit (Bioline). Microarray analysis using Affymetrix GeneChip® Human Gene 1.0 ST Arrays was performed on 4 biological replicates per cell line at the UNSW *Ramaciotti* Centre for Gene Expression Analysis. Microarray data was analysed using the R (http://www.R-project.org), Affy [50] and Limma packages [51]. Robust multi-array average (RMA) normalisation and background correction was used to remove any non-biological variations in the data, which was then fitted using a linear model. Contrasts were used to estimate differential expression and standard errors. Next, we filtered the data by applying a 2-fold change cut off and selecting genes with false discovery rate (fdr) adjusted p-values [52] of less than 0.05. RNA-Seq analysis was performed on 4 biological replicates per cell line using an Illumina HiSeq 2000 instrument (Illumina). The 50 bp paired-end reads generated were aligned to the genome using Bowtie2 [53] and the reads counted using HTSeq. The differential expression was then calculated utilizing the DESeq package [54].Heatmaps were generated using R package gplots. For real-time quantitative PCR (RT-qPCR), RNA was reverse transcribed using the ThermoScript RT-PCR system (Invitrogen) for first-strand cDNA synthesis. The cDNA was PCR-amplified in triplicate using the Fast SYBR green dye on the StepOnePlus Real-Time PCR system (Applied Biosystems). Relative expression was determined using BEshControl or BEsiGFP cells as reference samples, and GAPDH as an internal control. Sequences of primers used in this study are provided in Table S8 in File S1.

### ChIP and ChIP-Seq

Chromatin immunoprecipitation (ChIP) was performed as described previously [55]. For each ChIP assay, we incubated 25 µl of anti-FLAG M2 affinity beads (Sigma-Aldrich) overnight with cross-linked chromatin fragments from 1.5×10^7^ BE cells stably expressing pBABE-FLAG-FRA1 or empty vector (negative control). High-throughput sequencing of immunoprecipitated FLAG/StrepII-FRA1 and input chromatin was performed on an Illumina Genome Analyzer II (Illumina). We used 12.5 ng of ChIPed DNA to prepare sequencing libraries and sequenced two flow cell lanes per sample. Selected FRA1 targets were validated by ChIP followed by qPCR, with relative enrichment levels calculated after normalising against background enrichment determined in the negative control. The 36 bp ChIP-Seq reads were aligned to the human b37/hg19 reference genome using the Burrows-Wheeler Aligner (BWA) [56] and peaks were called using Model-based Analysis of ChIP-Seq (MACS) [57]. Input genomic sequences served as negative control. Statistical analysis of the resulting bam file and peaks was performed with the R package (http://www.R-project.org). The Bioconductor [58], Rsamtools and ChIPpeakAnno packages [59] were used to extract data from the bam files and to annotate the predicted peaks. Using custom scripts, the human genome was split into 1 kb bins and the number of tags in each bin calculated. Predicted peaks within 250 bp of each other were combined and each resulting peak matched with a bin. The number of tags at each summit was calculated and a normalised fold change, taking into account total reads, was calculated for the summit, peak and bin regions. Each peak was annotated using Ensembl GRCh37 version 61. Subsequently, motif analysis was performed using the MEME suite [60] and the data visualised with the Integrative Genomic Viewer (IGV 2.0) [61]. Comparison of ChIP-Seq reads near FRA1^EMT^ genes to a known AP-1 motif (MA0099.1, Jaspar core database) was performed using FIMO (MEME suite), with a p-value threshold less than 0.0001. Further data analysis was performed using the Galaxy platform [62]. The pie chart illustrating the location of ChIP-Seq reads relative to the transcription start site (TSS) of FRA1^EMT^ was generated using SoleSearch [63]. Gene cluster analysis of genes identified by microarray and ChIP-Seq was performed using GeneGo. EMT-related genes identified by GeneGo in both datasets were termed FRA1^EMT^ genes.

### Analysis of gene expression in human tumors

Previously published gene expression data were retrieved for primary colorectal cancers from 91 stage B and 94 stage C patients from the Royal Melbourne Hospital, Western Hospital, and Peter MacCallum Cancer Center in Australia, and the H. Lee Moffitt Cancer Center in the United States [12]. The median age at cancer diagnosis was 67 years (range 26–92 years); 98 patients were male and 87 were female. Follow-up and adjuvant treatment details were available from Biogrid Australia for Australian patients and the Moffitt Cancer Center Tumor Registry for U.S. patients. All samples had been analysed using HG-U133Plus2.0 GeneChip arrays (Affymetrix). Array data were RMA normalized and expression values log2 transformed. For unsupervised clustering, expression values for FRA1 silencing associated EMT genes were mean and sample centered followed by divisive hierarchical clustering using pair distances calculated as 1 minus the Spearman r as distance metric. Differences in mean gene expression values were calculated for the samples within the two main branches of the resulting dendrogram and assessed for statistical significance using the t-test with Benjamini and Hochberg multiple-testing correction. Relative upregulation or downregulation of gene expression between these two groups was assessed for consistency with upregulation or downregulation observed between FRA1 silencing and control cells using Pearson's χ^2^ test. All data processing and analysis were conducted using the statistical software package R and associated Bioconductor packages. Data processing and analysis were conducted using the statistical software package R. Differences between groups were assessed using the χ2 test for categorical variables and the Wilcoxon rank-sum test for continuous variables. For the outcome analysis, recurrence-free survival was defined as the time of surgery to the first confirmed relapse. Kaplan-Meier survival curves were generated using the PrognoScan algorithm [64]. Censoring was done when a patient died or was alive without recurrence at last contact. Cox proportional-hazards models were used to estimate survival distributions and hazard ratios, and were adjusted for patient characteristics as indicted. All statistical analyses were 2-sided and considered significant if P<0.05.

## Supporting Information

File S1 **Tables S1–S8.** Table S1. Relative changes in expression of FRA1 bound EMT-related genes upon FRA1 knockdown in BE CRC cells. Data represent means from 3 independent RNA-Seq experiments. Table S2. ChIP-Seq reads identified near FRA1^EMT^ genes. Table S3. AP-1 consensus motifs identified in FRA1^EMT^ genes. Table S4. Mean gene expression levels (log2) for the two main groups resulting from unsupervised clustering of stage B and C colorectal cancers using FRA1^EMT^ genes. Table S5. Clinicopathological and molecular associations for *FOSL1* expression levels in stage B and C CRC patients. Table S6. Clinicopathological and molecular associations for FRA1^EMT^ signature in stage B and C CRC patients. Table S7. Univariate and multivariate Cox proportional-hazards analysis of survival for stage B and C colorectal cancer patients according to *FOSL1* expression and the concordant FRA1^EMT^ gene expression patterns. Table S8. List of qRT-PCR and ChIP primers used in this study.
(PDF)
