# Supplementary material for: Widespread FRA1-Dependent Control of Mesenchymal Transdifferentiation Programs in Colorectal Cancer Cells
Source: PLoS One. 2014 Mar 21;9(3):e88950. doi: 10.1371/journal.pone.0088950 (PMC3962334; doi:10.1371/journal.pone.0088950)
Supplement: File S1 — Tables S1–S8. Table S1. Relative changes in expression of FRA1 bound EMT-related genes upon FRA1 knockdown in BE CRC cells. Data represent means from 3 independent RNA-Seq experiments. Table S2. ChIP-Seq reads identified near FRA1EMT genes. Table S3. AP-1 consensus motifs identified in FRA1EMT genes. Table S4. Mean gene expression levels (log2) for the two main groups resulting from unsupervised clustering of stage B and C colorectal cancers using FRA1EMT genes. Table S5. Clinicopathological and molecular associations for FOSL1 expression levels in stage B and C CRC patients. Table S6. Clinicopathological and molecular associations for FRA1EMT signature in stage B and C CRC patients. Table S7. Univariate and multivariate Cox proportional-hazards analysis of survival for stage B and C colorectal cancer patients according to FOSL1 expression and the concordant FRA1EMT gene expression patterns. Table S8. List of qRT-PCR and ChIP primers used in this study. (PDF) [file pone.0088950.s001.pdf]

Supporting Information

Widespread FRA1-dependent control of mesenchymal transdifferentiation programs in colorectal cancer cells

Jeannine Diesch[1,2], Elaine Sanij[1,3,4], Omer Gilan[1,2], Christopher Love[5], Hoanh Tran[6], Nicholas I. Fleming[5], Jason Ellul[1], Marcia Amalia[1,2], Izhak Haviv[7], Richard B. Pearson[1,2,3], Eugene Tulchinsky[8], John M. Mariadason[6], Oliver M. Sieber[5,11], Ross D. Hannan[1,2,3,9,10,11] and Amardeep S. Dhillon[1,3,4,11*]

[*]To whom correspondence should be addressed: Amardeep.Dhillon@petermac.org;
Ph: + 61 3 9656 1279; Fax: + 61 3 9656 1411

Table S1. Relative changes in expression of FRA1 bound EMT-related genes upon FRA1 knockdown in BE CRC cells. Data represent means from 3 independent RNA-Seq experiments.

Gene symbol	shFRA1-A1 vs shNS (log2)	shFRA1-A2 vs shNS (log2)
	**ADHESION**	
CLDN4	6.295790479	6.876151546
TPM2	2.525388332	2.760680361
CDH1	8.257600651	8.188627939
CLDN7	4.947907433	5.025261684
EPCAM	6.06897627	6.209767802
OLCN	1.844890865	1.752921708
TJP3	5.393108929	5.440078454
LPXN	-5.179435147	-5.195820698
PCDHB9	-5.53027117	-4.851388807
CDH4	-8.974351387	-8.314814613
TPM1	-2.721804346	-2.577430334
TJP2	-2.077037931	-1.952244558
	**SIGNALING**	
WNT16	9.836522059	10.77369936
FZD3	1.476620222	1.321437611
WNT10B	4.657953714	4.71506399
PDGFB	3.786449239	3.422630213
RAC3	3.131805551	3.219393614
NRP1	-7.666989679	-6.204335866
ROBO1	-9.19985079	-11.81316047
EGFR	-1.888197577	-2.98739624
RND3	-2.607296099	-3.120533259
RAC2	-2.390926409	-2.282730659
WNT5A	-10.05601808	-8.883612275
AXL	-4.98352569	-4.78509022
	**TRANSCRIPTION**	
SNAI2	-8.317520305	-11.02624484
LEF1	-9.326497653	-10.33717393
STAT3	-1.404080032	-1.451621599
JUN	-3.749516015	-2.9990092
STAT5B	-3.752912711	-3.503903836
ZEB1	-4.853994015	-4.823022834
ETS1	-2.727966164	-2.870310427

**ECM**		
RAB25	9.9954161	9.952912414
TNC	-10.84675053	-13.72765288
FBN1	-6.340193087	-6.597707995
LAMA3	-2.866431027	-2.790325669
ITGA6	-1.790897657	-1.917338348
ITGAV	-1.455695175	-1.142714962
ITGA3	-2.007918425	-1.889281641
LAMC1	-2.080575652	-1.692933554
SERPINE1	-4.230554369	-4.214337837
LAMC2	-3.314121432	-3.218130073
LAMB3	-3.285678298	-4.263050812
PLAU	-4.46943822	-3.577735794
MMP14	-3.473348296	-4.282728087
MMP16	-7.137263075	-10.52306725
LAMA4	-6.075175744	-5.049612234
ITGA2	-2.676398874	-3.165680981
ITGBL1	-4.787614888	-4.861324474
ITGA5	-1.549570822	-1.747777255
LAMB1	-3.004725793	-1.897362424
ITGB2	-5.290391571	-4.958685772
PLAUR	-3.170031299	-2.302493668
FN1	-7.845234765	-7.691191505
VIM	-10.00237061	-12.93717126
**TGFβ PATHWAY**		
CD109	9.034302717	8.57297631
BMP4	3.794933632	1.032242403
BMP7	9.010137623	8.831907176
TGFBI	6.67	6.71
SMAD3	-2.683074478	-2.324191377
TGFB2	-6.969617857	-6.847828316
TGFBR2	-4.189646407	-3.90078682
NOG	-4.53457067	-5.873693216

Table S2. ChIP-Seq reads identified near FRA1^{EMT} genes.

Chr	Start	End	Strand	Ensembl geneid	Gene symbol
7	73199513	73200397	+	ENSG00000189143	CLDN4
7	73205611	73206569	+	ENSG00000189143	CLDN4
7	73208087	73208769	+	ENSG00000189143	CLDN4
7	73211362	73212733	+	ENSG00000189143	CLDN4
7	73217903	73218582	+	ENSG00000189143	CLDN4
7	73222154	73223061	+	ENSG00000189143	CLDN4
7	73223264	73224294	+	ENSG00000189143	CLDN4
7	73224388	73225999	+	ENSG00000189143	CLDN4
7	73226088	73227210	+	ENSG00000189143	CLDN4
7	73231297	73232276	+	ENSG00000189143	CLDN4
7	73236162	73237197	+	ENSG00000189143	CLDN4
7	73239033	73241022	+	ENSG00000189143	CLDN4
7	73242759	73243464	+	ENSG00000189143	CLDN4
7	73245065	73246980	+	ENSG00000189143	CLDN4
6	74405521	74406286	+	ENSG00000156535	CD109
6	74413466	74414095	+	ENSG00000156535	CD109
6	74417743	74419203	+	ENSG00000156535	CD109
6	74427171	74428022	+	ENSG00000156535	CD109
6	74449184	74449818	+	ENSG00000156535	CD109
6	74469465	74470402	+	ENSG00000156535	CD109
6	74486009	74487080	+	ENSG00000156535	CD109
6	74488850	74489457	+	ENSG00000156535	CD109
6	74490567	74490972	+	ENSG00000156535	CD109
6	74495439	74495992	+	ENSG00000156535	CD109
6	74499024	74499852	+	ENSG00000156535	CD109
6	74545048	74546981	+	ENSG00000156535	CD109
6	74550211	74551188	+	ENSG00000156535	CD109
1	92133752	92134186	-	ENSG00000069702	TGFBR3
1	92142225	92143099	-	ENSG00000069702	TGFBR3
1	92149135	92149504	-	ENSG00000069702	TGFBR3
1	92194414	92195021	-	ENSG00000069702	TGFBR3
1	92205408	92206340	-	ENSG00000069702	TGFBR3
1	92233330	92233826	-	ENSG00000069702	TGFBR3
1	92245979	92246499	-	ENSG00000069702	TGFBR3
1	92267817	92268246	-	ENSG00000069702	TGFBR3
1	92321509	92322036	-	ENSG00000069702	TGFBR3

1	92323715	92324787	-	ENSG00000069702	TGFBR3
1	92338535	92339168	-	ENSG00000069702	TGFBR3
1	92339944	92340424	-	ENSG00000069702	TGFBR3
1	92347620	92348280	-	ENSG00000069702	TGFBR3
1	92350812	92352145	-	ENSG00000069702	TGFBR3
1	92363764	92364526	-	ENSG00000069702	TGFBR3
1	92370852	92371334	-	ENSG00000069702	TGFBR3
1	92376114	92376799	-	ENSG00000069702	TGFBR3
1	92383747	92384874	-	ENSG00000069702	TGFBR3
1	92385983	92387061	-	ENSG00000069702	TGFBR3
1	92388857	92389527	-	ENSG00000069702	TGFBR3
14	54403726	54404224	-	ENSG00000125378	BMP4
14	54412738	54413979	-	ENSG00000125378	BMP4
14	54416685	54417394	-	ENSG00000125378	BMP4
14	54420021	54421089	-	ENSG00000125378	BMP4
14	54422342	54423934	-	ENSG00000125378	BMP4
14	54429842	54430902	-	ENSG00000125378	BMP4
14	54433802	54434482	-	ENSG00000125378	BMP4
14	54436525	54437369	-	ENSG00000125378	BMP4
20	55731983	55732560	-	ENSG00000101144	BMP7
20	55732663	55733560	-	ENSG00000101144	BMP7
20	55735919	55737743	-	ENSG00000101144	BMP7
20	55739679	55740454	-	ENSG00000101144	BMP7
20	55740514	55742416	-	ENSG00000101144	BMP7
20	55748074	55748803	-	ENSG00000101144	BMP7
20	55752237	55754932	-	ENSG00000101144	BMP7
20	55755019	55755892	-	ENSG00000101144	BMP7
20	55756919	55757769	-	ENSG00000101144	BMP7
20	55764495	55766140	-	ENSG00000101144	BMP7
20	55769714	55771078	-	ENSG00000101144	BMP7
20	55772818	55775359	-	ENSG00000101144	BMP7
20	55778301	55778955	-	ENSG00000101144	BMP7
20	55782592	55783294	-	ENSG00000101144	BMP7
20	55798627	55800471	-	ENSG00000101144	BMP7
20	55800724	55802179	-	ENSG00000101144	BMP7
20	55818380	55819272	-	ENSG00000101144	BMP7
20	55825835	55826432	-	ENSG00000101144	BMP7
20	55839181	55843214	-	ENSG00000101144	BMP7
15	67357104	67359319	+	ENSG00000166949	SMAD3

15	67361934	67362493	+	ENSG00000166949	SMAD3
15	67365880	67366477	+	ENSG00000166949	SMAD3
15	67413343	67413932	+	ENSG00000166949	SMAD3
15	67442889	67444164	+	ENSG00000166949	SMAD3
15	67456949	67458256	+	ENSG00000166949	SMAD3
15	67459962	67460914	+	ENSG00000166949	SMAD3
15	67472098	67473284	+	ENSG00000166949	SMAD3
15	67473383	67474480	+	ENSG00000166949	SMAD3
15	67481507	67482193	+	ENSG00000166949	SMAD3
10	123205221	123205790	-	ENSG00000066468	FGFR2
2	47594236	47595256	+	ENSG00000119888	EPCAM
2	47595426	47598092	+	ENSG00000119888	EPCAM
2	47599316	47600400	+	ENSG00000119888	EPCAM
2	47609917	47611160	+	ENSG00000119888	EPCAM
2	47611175	47612227	+	ENSG00000119888	EPCAM
2	47614041	47615170	+	ENSG00000119888	EPCAM
2	47617276	47618060	+	ENSG00000119888	EPCAM
19	41720206	41721304	+	ENSG00000167601	AXL
19	41724438	41726773	+	ENSG00000167601	AXL
19	41727514	41728773	+	ENSG00000167601	AXL
19	41729222	41730350	+	ENSG00000167601	AXL
19	41731457	41733185	+	ENSG00000167601	AXL
19	41742898	41747468	+	ENSG00000167601	AXL
19	41754998	41755842	+	ENSG00000167601	AXL
19	41756000	41757682	+	ENSG00000167601	AXL
19	41759711	41760622	+	ENSG00000167601	AXL
19	41761094	41761950	+	ENSG00000167601	AXL
19	41765359	41766591	+	ENSG00000167601	AXL
5	135339438	135339942	+	ENSG00000120708	TGFBI
5	135364095	135365544	+	ENSG00000120708	TGFBI
5	135381904	135382275	+	ENSG00000120708	TGFBI
5	135402462	135402939	+	ENSG00000120708	TGFBI
11	58299067	58300192	-	ENSG00000110031	LPXN
11	58321364	58321992	-	ENSG00000110031	LPXN
15	48817164	48817746	-	ENSG00000166147	FBN1
15	48870559	48871200	-	ENSG00000166147	FBN1
15	48897682	48898177	-	ENSG00000166147	FBN1
15	48903305	48903908	-	ENSG00000166147	FBN1
15	48905689	48906249	-	ENSG00000166147	FBN1

15	48936795	48938624	-	ENSG00000166147	FBN1
1	183144103	183144720	+	ENSG00000058085	LAMC2
1	183150523	183151322	+	ENSG00000058085	LAMC2
1	183154874	183155698	+	ENSG00000058085	LAMC2
9	117839117	117839894	-	ENSG00000041982	TNC
9	117847910	117849615	-	ENSG00000041982	TNC
9	117852669	117853490	-	ENSG00000041982	TNC
1	209790660	209792394	-	ENSG00000196878	LAMB3
1	209795578	209797515	-	ENSG00000196878	LAMB3
1	209798755	209799499	-	ENSG00000196878	LAMB3
1	209799815	209801939	-	ENSG00000196878	LAMB3
1	209803805	209804687	-	ENSG00000196878	LAMB3
1	209807401	209808621	-	ENSG00000196878	LAMB3
10	75654955	75656684	+	ENSG00000122861	PLAU
10	75670520	75671907	+	ENSG00000122861	PLAU
19	44148760	44150146	-	ENSG00000011422	PLAUR
19	44150851	44151252	-	ENSG00000011422	PLAUR
19	44154162	44154771	-	ENSG00000011422	PLAUR
19	44155007	44156198	-	ENSG00000011422	PLAUR
19	44158351	44159434	-	ENSG00000011422	PLAUR
19	44159851	44161729	-	ENSG00000011422	PLAUR
19	44169018	44170360	-	ENSG00000011422	PLAUR
19	44171528	44172282	-	ENSG00000011422	PLAUR
19	44172654	44174633	-	ENSG00000011422	PLAUR
19	44179710	44180671	-	ENSG00000011422	PLAUR
19	44181667	44182392	-	ENSG00000011422	PLAUR
19	44184971	44185664	-	ENSG00000011422	PLAUR
19	44186555	44187139	-	ENSG00000011422	PLAUR
19	44191484	44192232	-	ENSG00000011422	PLAUR
3	30629847	30630379	+	ENSG00000163513	TGFBR2
3	30632693	30633410	+	ENSG00000163513	TGFBR2
3	30647769	30649022	+	ENSG00000163513	TGFBR2
3	30649347	30649833	+	ENSG00000163513	TGFBR2
3	30659570	30660143	+	ENSG00000163513	TGFBR2
3	30678073	30678673	+	ENSG00000163513	TGFBR2
3	30694580	30695177	+	ENSG00000163513	TGFBR2
3	30719570	30720120	+	ENSG00000163513	TGFBR2
6	112445804	112446321	-	ENSG00000112769	LAMA4
6	112457500	112457989	-	ENSG00000112769	LAMA4

6	112502859	112503323	-	ENSG00000112769	LAMA4
6	112511904	112512554	-	ENSG00000112769	LAMA4
6	112532951	112533767	-	ENSG00000112769	LAMA4
11	127845612	127846709	-	ENSG00000134954	ETS1
11	127868765	127869322	-	ENSG00000134954	ETS1
11	127902786	127903385	-	ENSG00000134954	ETS1
11	128009832	128010536	-	ENSG00000134954	ETS1
11	128084686	128085854	-	ENSG00000134954	ETS1
11	128092781	128093738	-	ENSG00000134954	ETS1
11	128106707	128107738	-	ENSG00000134954	ETS1
11	128111614	128112048	-	ENSG00000134954	ETS1
11	128189066	128189513	-	ENSG00000134954	ETS1
11	128210432	128210950	-	ENSG00000134954	ETS1
11	128292414	128292968	-	ENSG00000134954	ETS1
11	128295389	128296054	-	ENSG00000134954	ETS1
11	128345300	128345785	-	ENSG00000134954	ETS1
11	128428073	128428600	-	ENSG00000134954	ETS1
11	128489281	128489839	-	ENSG00000134954	ETS1
11	128502289	128502751	-	ENSG00000134954	ETS1
17	40351455	40352115	-	ENSG00000173757	STAT5B
17	40353736	40354783	-	ENSG00000173757	STAT5B
17	40357754	40359166	-	ENSG00000173757	STAT5B
17	40359624	40361287	-	ENSG00000173757	STAT5B
17	40365169	40366160	-	ENSG00000173757	STAT5B
17	40367532	40368244	-	ENSG00000173757	STAT5B
17	40369478	40372404	-	ENSG00000173757	STAT5B
17	40372472	40373402	-	ENSG00000173757	STAT5B
17	40373470	40374411	-	ENSG00000173757	STAT5B
17	40378377	40379405	-	ENSG00000173757	STAT5B
17	40380238	40381016	-	ENSG00000173757	STAT5B
17	40385393	40385817	-	ENSG00000173757	STAT5B
17	40387330	40387887	-	ENSG00000173757	STAT5B
17	40388406	40388913	-	ENSG00000173757	STAT5B
17	40389652	40390744	-	ENSG00000173757	STAT5B
17	40396980	40398123	-	ENSG00000173757	STAT5B
17	40427479	40429191	-	ENSG00000173757	STAT5B
7	107581975	107582783	-	ENSG00000091136	LAMB1
7	107592031	107592683	-	ENSG00000091136	LAMB1
7	107594676	107595260	-	ENSG00000091136	LAMB1

7	107596039	107596760	-	ENSG00000091136	LAMB1
7	107597434	107598040	-	ENSG00000091136	LAMB1
7	107606310	107606955	-	ENSG00000091136	LAMB1
7	107617187	107617741	-	ENSG00000091136	LAMB1
7	107621316	107621880	-	ENSG00000091136	LAMB1
7	107625760	107626461	-	ENSG00000091136	LAMB1
7	107633466	107634008	-	ENSG00000091136	LAMB1
7	107641691	107642777	-	ENSG00000091136	LAMB1
7	107643656	107644061	-	ENSG00000091136	LAMB1
7	107651702	107652205	-	ENSG00000091136	LAMB1
12	54798618	54799559	-	ENSG00000161638	ITGA5
12	54801822	54804196	-	ENSG00000161638	ITGA5
12	54808188	54808933	-	ENSG00000161638	ITGA5
12	54809419	54810150	-	ENSG00000161638	ITGA5
12	54812076	54813318	-	ENSG00000161638	ITGA5
12	54827481	54828315	-	ENSG00000161638	ITGA5
12	54828391	54828844	-	ENSG00000161638	ITGA5
17	48137179	48137953	+	ENSG00000005884	ITGA3
17	48138240	48140311	+	ENSG00000005884	ITGA3
17	48141023	48142621	+	ENSG00000005884	ITGA3
17	48145081	48146768	+	ENSG00000005884	ITGA3
17	48149389	48150257	+	ENSG00000005884	ITGA3
17	48150674	48152312	+	ENSG00000005884	ITGA3
17	48152600	48153407	+	ENSG00000005884	ITGA3
17	48153412	48155158	+	ENSG00000005884	ITGA3
17	48156049	48158040	+	ENSG00000005884	ITGA3
17	48159160	48160096	+	ENSG00000005884	ITGA3
17	48163658	48164472	+	ENSG00000005884	ITGA3
17	48164650	48165987	+	ENSG00000005884	ITGA3
17	48166030	48168371	+	ENSG00000005884	ITGA3
1	182985051	182985788	+	ENSG00000135862	LAMC1
1	182986464	182986852	+	ENSG00000135862	LAMC1
1	182989367	182990281	+	ENSG00000135862	LAMC1
1	182991803	182993820	+	ENSG00000135862	LAMC1
1	183003256	183003794	+	ENSG00000135862	LAMC1
1	183005245	183006209	+	ENSG00000135862	LAMC1
1	183019261	183020089	+	ENSG00000135862	LAMC1
1	183022728	183023166	+	ENSG00000135862	LAMC1
1	183047286	183047709	+	ENSG00000135862	LAMC1

1	183056793	183057350	+	ENSG00000135862	LAMC1
1	183080066	183080591	+	ENSG00000135862	LAMC1
1	183084831	183085760	+	ENSG00000135862	LAMC1
1	183087429	183088369	+	ENSG00000135862	LAMC1
1	183118338	183119585	+	ENSG00000135862	LAMC1
9	71751987	71752723	+	ENSG00000119139	TJP2
9	71764918	71765624	+	ENSG00000119139	TJP2
9	71788712	71789592	+	ENSG00000119139	TJP2
9	71794262	71794728	+	ENSG00000119139	TJP2
9	71806504	71807228	+	ENSG00000119139	TJP2
9	71821207	71821946	+	ENSG00000119139	TJP2
9	71823971	71824692	+	ENSG00000119139	TJP2
9	71827932	71828360	+	ENSG00000119139	TJP2
9	71833698	71834274	+	ENSG00000119139	TJP2
9	71835705	71837562	+	ENSG00000119139	TJP2
9	71837577	71838598	+	ENSG00000119139	TJP2
9	71850060	71850655	+	ENSG00000119139	TJP2
9	71868864	71869563	+	ENSG00000119139	TJP2
9	71873651	71874377	+	ENSG00000119139	TJP2
9	71878681	71879334	+	ENSG00000119139	TJP2
9	71879888	71880386	+	ENSG00000119139	TJP2
9	71883765	71884266	+	ENSG00000119139	TJP2
9	71889784	71890312	+	ENSG00000119139	TJP2
9	71891993	71893460	+	ENSG00000119139	TJP2
9	71896680	71897503	+	ENSG00000119139	TJP2
2	187431364	187431949	+	ENSG00000138448	ITGAV
2	187436040	187436560	+	ENSG00000138448	ITGAV
2	187438843	187439403	+	ENSG00000138448	ITGAV
2	187451700	187452409	+	ENSG00000138448	ITGAV
2	187452895	187453551	+	ENSG00000138448	ITGAV
2	187454340	187455426	+	ENSG00000138448	ITGAV
2	187467172	187467696	+	ENSG00000138448	ITGAV
2	187480872	187481435	+	ENSG00000138448	ITGAV
2	187510051	187510888	+	ENSG00000138448	ITGAV
2	187513122	187513541	+	ENSG00000138448	ITGAV
2	187517493	187518103	+	ENSG00000138448	ITGAV
2	187536509	187537024	+	ENSG00000138448	ITGAV
2	187538272	187538977	+	ENSG00000138448	ITGAV
17	40466779	40467320	-	ENSG00000168610	STAT3

17	40481987	40483006	-	ENSG00000168610	STAT3
17	40483616	40484599	-	ENSG00000168610	STAT3
17	40490877	40491340	-	ENSG00000168610	STAT3
17	40491846	40492494	-	ENSG00000168610	STAT3
17	40496005	40497413	-	ENSG00000168610	STAT3
17	40506054	40506789	-	ENSG00000168610	STAT3
17	40518830	40519709	-	ENSG00000168610	STAT3
17	40521610	40522899	-	ENSG00000168610	STAT3
17	40523254	40524055	-	ENSG00000168610	STAT3
17	40529876	40530655	-	ENSG00000168610	STAT3
17	40531922	40533007	-	ENSG00000168610	STAT3
17	40534533	40535675	-	ENSG00000168610	STAT3
17	40535849	40536979	-	ENSG00000168610	STAT3
17	40539563	40540999	-	ENSG00000168610	STAT3
18	21269174	21270329	+	ENSG00000053747	LAMA3
18	21278018	21278587	+	ENSG00000053747	LAMA3
18	21320688	21321167	+	ENSG00000053747	LAMA3
18	21321682	21322210	+	ENSG00000053747	LAMA3
18	21341937	21343157	+	ENSG00000053747	LAMA3
18	21356583	21357112	+	ENSG00000053747	LAMA3
18	21370705	21371399	+	ENSG00000053747	LAMA3
18	21391423	21392299	+	ENSG00000053747	LAMA3
18	21422285	21422642	+	ENSG00000053747	LAMA3
18	21474670	21475138	+	ENSG00000053747	LAMA3
18	21499497	21499927	+	ENSG00000053747	LAMA3
18	21504067	21504794	+	ENSG00000053747	LAMA3
18	21520555	21521414	+	ENSG00000053747	LAMA3
18	21524768	21525481	+	ENSG00000053747	LAMA3
3	78631839	78633556	-	ENSG00000169855	ROBO1
3	78729316	78730529	-	ENSG00000169855	ROBO1
3	78760266	78760907	-	ENSG00000169855	ROBO1
3	78764608	78765138	-	ENSG00000169855	ROBO1
3	78988344	78988804	-	ENSG00000169855	ROBO1
3	79012384	79012965	-	ENSG00000169855	ROBO1
3	79067788	79068952	-	ENSG00000169855	ROBO1
3	79070668	79071269	-	ENSG00000169855	ROBO1
3	79138988	79139485	-	ENSG00000169855	ROBO1
3	79168844	79169338	-	ENSG00000169855	ROBO1
3	79179795	79180596	-	ENSG00000169855	ROBO1

3	79182129	79182751	-	ENSG00000169855	ROBO1
3	79215594	79216056	-	ENSG00000169855	ROBO1
3	79222223	79223224	-	ENSG00000169855	ROBO1
3	79246120	79247008	-	ENSG00000169855	ROBO1
3	79257679	79258131	-	ENSG00000169855	ROBO1
3	79286212	79286725	-	ENSG00000169855	ROBO1
3	79332690	79333315	-	ENSG00000169855	ROBO1
3	79339110	79339540	-	ENSG00000169855	ROBO1
3	79349075	79350113	-	ENSG00000169855	ROBO1
3	79372070	79372562	-	ENSG00000169855	ROBO1
3	79412990	79413411	-	ENSG00000169855	ROBO1
3	79416927	79417412	-	ENSG00000169855	ROBO1
3	79421243	79421787	-	ENSG00000169855	ROBO1
3	79430020	79430683	-	ENSG00000169855	ROBO1
3	79494505	79495333	-	ENSG00000169855	ROBO1
3	79545241	79545946	-	ENSG00000169855	ROBO1
3	79549912	79550283	-	ENSG00000169855	ROBO1
3	79551136	79552160	-	ENSG00000169855	ROBO1
3	79555455	79555964	-	ENSG00000169855	ROBO1
3	79568340	79568853	-	ENSG00000169855	ROBO1
3	79660461	79661927	-	ENSG00000169855	ROBO1
3	79733728	79734153	-	ENSG00000169855	ROBO1
3	79769839	79770485	-	ENSG00000169855	ROBO1
3	79774222	79774780	-	ENSG00000169855	ROBO1
3	79815450	79816720	-	ENSG00000169855	ROBO1
3	79834126	79834656	-	ENSG00000169855	ROBO1
3	79863606	79864372	-	ENSG00000169855	ROBO1
3	79873165	79873836	-	ENSG00000169855	ROBO1
10	33460696	33461147	-	ENSG00000099250	NRP1
10	33477323	33477830	-	ENSG00000099250	NRP1
10	33492500	33492925	-	ENSG00000099250	NRP1
10	33493529	33493994	-	ENSG00000099250	NRP1
10	33497676	33498114	-	ENSG00000099250	NRP1
10	33504877	33505709	-	ENSG00000099250	NRP1
10	33559098	33559672	-	ENSG00000099250	NRP1
10	33566249	33566607	-	ENSG00000099250	NRP1
10	33623035	33626337	-	ENSG00000099250	NRP1
10	33675991	33676937	-	ENSG00000099250	NRP1
10	33705638	33706045	-	ENSG00000099250	NRP1

10	33707507	33708112	-	ENSG00000099250	NRP1
10	33709071	33709652	-	ENSG00000099250	NRP1
10	33727722	33728371	-	ENSG00000099250	NRP1
10	33730971	33731871	-	ENSG00000099250	NRP1
14	23305534	23307093	+	ENSG00000157227	MMP14
14	23309441	23310355	+	ENSG00000157227	MMP14
14	23311258	23312029	+	ENSG00000157227	MMP14
14	23314892	23315623	+	ENSG00000157227	MMP14
14	23315741	23316679	+	ENSG00000157227	MMP14
14	23317640	23318273	+	ENSG00000157227	MMP14
8	89092501	89092972	-	ENSG00000156103	MMP16
8	89161038	89162177	-	ENSG00000156103	MMP16
8	89163070	89163968	-	ENSG00000156103	MMP16
8	89169878	89171040	-	ENSG00000156103	MMP16
8	89339501	89340541	-	ENSG00000156103	MMP16
10	17270013	17272890	+	ENSG00000026025	VIM
10	17275993	17276623	+	ENSG00000026025	VIM
10	17314436	17315186	+	ENSG00000026025	VIM
2	216250354	216251240	-	ENSG00000115414	FN1
2	216276303	216276803	-	ENSG00000115414	FN1
2	216289932	216291190	-	ENSG00000115414	FN1
2	216291471	216291940	-	ENSG00000115414	FN1
2	216300198	216300946	-	ENSG00000115414	FN1
8	49835794	49836343	-	ENSG00000019549	SNAI2
8	49880992	49881640	-	ENSG00000019549	SNAI2
8	49889581	49890269	-	ENSG00000019549	SNAI2
1	218541048	218541884	+	ENSG00000092969	TGFB2
1	218551228	218552131	+	ENSG00000092969	TGFB2
1	218568619	218569323	+	ENSG00000092969	TGFB2
1	218586285	218586829	+	ENSG00000092969	TGFB2
1	218600533	218602516	+	ENSG00000092969	TGFB2
1	218628539	218629602	+	ENSG00000092969	TGFB2
4	108977908	108978404	-	ENSG00000138795	LEF1
4	108978982	108979370	-	ENSG00000138795	LEF1
4	108998099	108999116	-	ENSG00000138795	LEF1
4	109001926	109002908	-	ENSG00000138795	LEF1
4	109045040	109045877	-	ENSG00000138795	LEF1
4	109053708	109054406	-	ENSG00000138795	LEF1
4	109087613	109090536	-	ENSG00000138795	LEF1

5	52284656	52285880	+	ENSG00000164171	ITGA2
10	31619909	31620397	+	ENSG00000148516	ZEB1
10	31628289	31628797	+	ENSG00000148516	ZEB1
10	31694566	31695307	+	ENSG00000148516	ZEB1
10	31695899	31697118	+	ENSG00000148516	ZEB1
10	31730493	31731220	+	ENSG00000148516	ZEB1
10	31732421	31733742	+	ENSG00000148516	ZEB1
10	31737435	31738398	+	ENSG00000148516	ZEB1
10	31796881	31797956	+	ENSG00000148516	ZEB1
10	31828312	31828727	+	ENSG00000148516	ZEB1
17	45320806	45322021	+	ENSG00000056345	ITGB3
17	45327484	45328259	+	ENSG00000056345	ITGB3
17	45330706	45333349	+	ENSG00000056345	ITGB3
17	45352577	45353465	+	ENSG00000056345	ITGB3
17	45357550	45358415	+	ENSG00000056345	ITGB3
17	45369368	45370040	+	ENSG00000056345	ITGB3
17	45376182	45377219	+	ENSG00000056345	ITGB3
17	45390242	45391095	+	ENSG00000056345	ITGB3
17	45391127	45391768	+	ENSG00000056345	ITGB3
13	102104875	102105369	+	ENSG00000198542	ITGBL1
13	102105808	102106847	+	ENSG00000198542	ITGBL1
13	102121617	102122072	+	ENSG00000198542	ITGBL1
13	102184013	102184449	+	ENSG00000198542	ITGBL1
13	102191242	102193168	+	ENSG00000198542	ITGBL1
13	102208540	102208995	+	ENSG00000198542	ITGBL1
13	102330723	102331675	+	ENSG00000198542	ITGBL1
13	102333868	102334395	+	ENSG00000198542	ITGBL1
13	102361268	102361836	+	ENSG00000198542	ITGBL1
22	37621548	37624023	-	ENSG00000128340	RAC2
22	37624414	37626185	-	ENSG00000128340	RAC2
22	37626978	37628247	-	ENSG00000128340	RAC2
22	37629185	37630949	-	ENSG00000128340	RAC2
22	37631303	37631783	-	ENSG00000128340	RAC2
22	37632777	37634892	-	ENSG00000128340	RAC2
22	37636800	37638057	-	ENSG00000128340	RAC2
22	37638708	37639672	-	ENSG00000128340	RAC2
22	37639711	37641290	-	ENSG00000128340	RAC2
22	37643506	37644035	-	ENSG00000128340	RAC2
22	37646961	37647431	-	ENSG00000128340	RAC2

22	37649476	37650603	-	ENSG00000128340	RAC2
22	37651513	37652930	-	ENSG00000128340	RAC2
2	151278081	151278520	-	ENSG00000115963	RND3
2	151341645	151343563	-	ENSG00000115963	RND3
2	151349839	151350277	-	ENSG00000115963	RND3
2	151357787	151358335	-	ENSG00000115963	RND3
2	151369097	151369739	-	ENSG00000115963	RND3
2	151372747	151373253	-	ENSG00000115963	RND3
2	151386938	151387527	-	ENSG00000115963	RND3
2	151390320	151390802	-	ENSG00000115963	RND3
2	151399130	151399743	-	ENSG00000115963	RND3
2	151399787	151400290	-	ENSG00000115963	RND3
15	63309435	63310044	+	ENSG00000140416	TPM1
15	63325084	63325678	+	ENSG00000140416	TPM1
15	63333918	63336377	+	ENSG00000140416	TPM1
15	63340144	63341436	+	ENSG00000140416	TPM1
15	63345735	63346259	+	ENSG00000140416	TPM1
15	63376065	63376939	+	ENSG00000140416	TPM1
15	63379337	63380213	+	ENSG00000140416	TPM1
20	59823031	59823842	+	ENSG00000179242	CDH4
20	59826752	59829928	+	ENSG00000179242	CDH4
20	59831471	59832061	+	ENSG00000179242	CDH4
20	59854615	59857168	+	ENSG00000179242	CDH4
20	59857426	59859217	+	ENSG00000179242	CDH4
20	59862223	59863039	+	ENSG00000179242	CDH4
20	59863276	59864817	+	ENSG00000179242	CDH4
20	59867282	59870225	+	ENSG00000179242	CDH4
20	60318398	60320287	+	ENSG00000179242	CDH4
20	60320566	60322646	+	ENSG00000179242	CDH4
20	60338989	60343417	+	ENSG00000179242	CDH4
20	60345057	60346661	+	ENSG00000179242	CDH4
20	60347849	60348848	+	ENSG00000179242	CDH4
20	60359531	60360980	+	ENSG00000179242	CDH4
20	60364244	60366220	+	ENSG00000179242	CDH4
20	60366547	60369235	+	ENSG00000179242	CDH4
20	60370707	60371828	+	ENSG00000179242	CDH4
20	60371850	60374148	+	ENSG00000179242	CDH4
20	60374406	60377225	+	ENSG00000179242	CDH4
20	60377464	60379886	+	ENSG00000179242	CDH4

20	60380236	60383421	+	ENSG00000179242	CDH4
20	60383569	60384557	+	ENSG00000179242	CDH4
20	60384612	60386312	+	ENSG00000179242	CDH4
20	60386528	60387633	+	ENSG00000179242	CDH4
20	60387667	60390691	+	ENSG00000179242	CDH4
20	60391509	60392046	+	ENSG00000179242	CDH4
20	60392146	60393315	+	ENSG00000179242	CDH4
20	60393494	60394819	+	ENSG00000179242	CDH4
20	60394884	60396750	+	ENSG00000179242	CDH4
20	60403717	60406308	+	ENSG00000179242	CDH4
20	60408994	60410220	+	ENSG00000179242	CDH4
20	60410257	60411320	+	ENSG00000179242	CDH4
20	60411930	60413795	+	ENSG00000179242	CDH4
20	60419594	60423976	+	ENSG00000179242	CDH4
20	60424237	60425318	+	ENSG00000179242	CDH4
20	60425579	60429039	+	ENSG00000179242	CDH4
20	60429048	60431247	+	ENSG00000179242	CDH4
20	60434695	60435266	+	ENSG00000179242	CDH4
20	60435456	60437813	+	ENSG00000179242	CDH4
20	60438163	60440489	+	ENSG00000179242	CDH4
20	60442888	60445884	+	ENSG00000179242	CDH4
20	60446981	60451872	+	ENSG00000179242	CDH4
20	60451918	60455136	+	ENSG00000179242	CDH4
20	60455165	60460578	+	ENSG00000179242	CDH4
20	60460596	60462406	+	ENSG00000179242	CDH4
20	60462775	60467065	+	ENSG00000179242	CDH4
20	60467513	60469287	+	ENSG00000179242	CDH4
20	60469644	60472112	+	ENSG00000179242	CDH4
20	60472209	60476157	+	ENSG00000179242	CDH4
20	60476195	60477758	+	ENSG00000179242	CDH4
20	60478568	60480227	+	ENSG00000179242	CDH4
20	60480370	60480825	+	ENSG00000179242	CDH4
20	60480892	60483282	+	ENSG00000179242	CDH4
20	60483314	60484792	+	ENSG00000179242	CDH4
20	60484869	60486039	+	ENSG00000179242	CDH4
20	60487821	60489420	+	ENSG00000179242	CDH4
20	60489754	60490894	+	ENSG00000179242	CDH4
20	60497491	60503886	+	ENSG00000179242	CDH4
20	60504280	60510622	+	ENSG00000179242	CDH4

20	60510661	60512351	+	ENSG00000179242	CDH4
20	60512552	60514751	+	ENSG00000179242	CDH4
2	173248534	173249024	+	ENSG00000091409	ITGA6
2	173282086	173282689	+	ENSG00000091409	ITGA6
2	173288260	173288735	+	ENSG00000091409	ITGA6
2	173291898	173293161	+	ENSG00000091409	ITGA6
5	140568055	140569190	+	ENSG00000177839	PCDHB9
9	101866553	101868409	+	ENSG00000106799	TGFBR1
9	101921480	101922177	+	ENSG00000106799	TGFBR1
9	101923985	101924597	+	ENSG00000106799	TGFBR1
1	156029632	156031210	+	ENSG00000132698	RAB25
1	156035595	156036227	+	ENSG00000132698	RAB25
1	156036288	156037009	+	ENSG00000132698	RAB25
1	156037319	156039431	+	ENSG00000132698	RAB25
1	156039558	156041079	+	ENSG00000132698	RAB25
17	7163994	7166613	-	ENSG00000181885	CLDN7
17	7166712	7167376	-	ENSG00000181885	CLDN7
16	68757400	68758228	+	ENSG00000039068	CDH1
16	68759099	68759837	+	ENSG00000039068	CDH1
16	68761789	68762608	+	ENSG00000039068	CDH1
16	68763352	68764449	+	ENSG00000039068	CDH1
16	68769167	68772534	+	ENSG00000039068	CDH1
16	68811246	68812334	+	ENSG00000039068	CDH1
16	68821275	68822112	+	ENSG00000039068	CDH1
16	68828377	68828990	+	ENSG00000039068	CDH1
16	68829110	68830152	+	ENSG00000039068	CDH1
16	68838640	68840457	+	ENSG00000039068	CDH1
16	68847351	68848424	+	ENSG00000039068	CDH1
16	68852138	68853193	+	ENSG00000039068	CDH1
16	68857741	68858359	+	ENSG00000039068	CDH1
16	68870690	68871962	+	ENSG00000039068	CDH1
9	35682527	35683372	-	ENSG00000198467	TPM2
9	35684500	35685649	-	ENSG00000198467	TPM2
9	35689139	35691700	-	ENSG00000198467	TPM2
9	35692696	35693749	-	ENSG00000198467	TPM2
17	79989492	79992990	+	ENSG00000169750	RAC3
19	3717741	3718343	+	ENSG00000105289	TJP3
19	3720647	3721396	+	ENSG00000105289	TJP3
19	3721398	3722269	+	ENSG00000105289	TJP3

19	3724064	3724847	+	ENSG00000105289	TJP3
19	3727657	3729180	+	ENSG00000105289	TJP3
19	3729312	3731188	+	ENSG00000105289	TJP3
19	3732526	3733418	+	ENSG00000105289	TJP3
19	3733751	3735498	+	ENSG00000105289	TJP3
19	3735838	3737244	+	ENSG00000105289	TJP3
19	3738325	3739585	+	ENSG00000105289	TJP3
19	3741008	3741827	+	ENSG00000105289	TJP3
19	3742478	3743240	+	ENSG00000105289	TJP3
19	3744275	3745462	+	ENSG00000105289	TJP3
19	3746076	3748545	+	ENSG00000105289	TJP3
19	3750537	3755032	+	ENSG00000105289	TJP3
7	55047639	55048090	+	ENSG00000146648	EGFR
7	55071433	55073479	+	ENSG00000146648	EGFR
7	55086020	55088310	+	ENSG00000146648	EGFR
7	55123241	55123682	+	ENSG00000146648	EGFR
7	55145508	55147231	+	ENSG00000146648	EGFR
7	55163191	55163806	+	ENSG00000146648	EGFR
7	55189887	55190448	+	ENSG00000146648	EGFR
7	55198346	55198822	+	ENSG00000146648	EGFR
7	55201158	55202229	+	ENSG00000146648	EGFR
7	55211997	55213112	+	ENSG00000146648	EGFR
7	55219028	55219762	+	ENSG00000146648	EGFR
7	55220726	55221155	+	ENSG00000146648	EGFR
7	55221543	55222285	+	ENSG00000146648	EGFR
7	55228582	55229392	+	ENSG00000146648	EGFR
7	55234129	55235562	+	ENSG00000146648	EGFR
7	55236691	55237133	+	ENSG00000146648	EGFR
7	55245005	55246565	+	ENSG00000146648	EGFR
7	55259820	55260450	+	ENSG00000146648	EGFR
7	55270639	55271618	+	ENSG00000146648	EGFR
7	55271739	55273106	+	ENSG00000146648	EGFR
7	55287001	55289015	+	ENSG00000146648	EGFR
7	55290850	55291591	+	ENSG00000146648	EGFR
7	55318364	55318910	+	ENSG00000146648	EGFR
7	55321971	55323179	+	ENSG00000146648	EGFR
7	55325216	55325889	+	ENSG00000146648	EGFR
8	28351140	28352474	+	ENSG00000104290	FZD3
8	28358172	28358624	+	ENSG00000104290	FZD3

8	28400745	28401309	+	ENSG00000104290	FZD3
8	28405277	28405766	+	ENSG00000104290	FZD3
8	28418454	28418874	+	ENSG00000104290	FZD3
1	59242032	59242625	-	ENSG00000177606	JUN
1	59247591	59251299	-	ENSG00000177606	JUN
5	68788039	68790332	+	ENSG00000197822	OCLN
5	68803812	68804585	+	ENSG00000197822	OCLN
5	68810501	68812531	+	ENSG00000197822	OCLN
5	68812939	68813458	+	ENSG00000197822	OCLN
5	68829365	68829844	+	ENSG00000197822	OCLN
5	68832094	68833204	+	ENSG00000197822	OCLN
5	68844010	68844794	+	ENSG00000197822	OCLN
5	68847722	68848463	+	ENSG00000197822	OCLN
5	68850668	68851406	+	ENSG00000197822	OCLN
22	39594848	39595349	-	ENSG00000100311	PDGFB
22	39596317	39597020	-	ENSG00000100311	PDGFB
22	39600659	39601842	-	ENSG00000100311	PDGFB
22	39602988	39604612	-	ENSG00000100311	PDGFB
22	39605505	39606985	-	ENSG00000100311	PDGFB
22	39609142	39609900	-	ENSG00000100311	PDGFB
22	39615898	39616989	-	ENSG00000100311	PDGFB
22	39619070	39619731	-	ENSG00000100311	PDGFB
22	39620393	39621094	-	ENSG00000100311	PDGFB
22	39621379	39622188	-	ENSG00000100311	PDGFB
22	39622646	39623516	-	ENSG00000100311	PDGFB
22	39625971	39627127	-	ENSG00000100311	PDGFB
22	39627269	39628192	-	ENSG00000100311	PDGFB
22	39629335	39629979	-	ENSG00000100311	PDGFB
22	39631026	39632094	-	ENSG00000100311	PDGFB
22	39633554	39634410	-	ENSG00000100311	PDGFB
22	39636973	39642244	-	ENSG00000100311	PDGFB
22	39645643	39646742	-	ENSG00000100311	PDGFB
22	39648764	39649292	-	ENSG00000100311	PDGFB
22	39649354	39651455	-	ENSG00000100311	PDGFB
22	39651990	39654451	-	ENSG00000100311	PDGFB
22	39654638	39655737	-	ENSG00000100311	PDGFB
22	39656395	39657251	-	ENSG00000100311	PDGFB
22	39658149	39658879	-	ENSG00000100311	PDGFB
22	39659967	39660963	-	ENSG00000100311	PDGFB

22	39661258	39662312	-	ENSG00000100311	PDGFB
22	39663034	39663721	-	ENSG00000100311	PDGFB
7	100753566	100754201	+	ENSG00000106366	SERPINE1
7	100757870	100758882	+	ENSG00000106366	SERPINE1
7	100760328	100761451	+	ENSG00000106366	SERPINE1
7	100765313	100765878	+	ENSG00000106366	SERPINE1
7	100768299	100768843	+	ENSG00000106366	SERPINE1
7	100779045	100779643	+	ENSG00000106366	SERPINE1
7	100785682	100786435	+	ENSG00000106366	SERPINE1
12	49359736	49360430	-	ENSG00000169884	WNT10B
12	49360648	49361505	-	ENSG00000169884	WNT10B
12	49361683	49362161	-	ENSG00000169884	WNT10B
12	49363500	49364708	-	ENSG00000169884	WNT10B
12	49365192	49366224	-	ENSG00000169884	WNT10B
12	49366228	49366869	-	ENSG00000169884	WNT10B
7	120968721	120969948	+	ENSG00000002745	WNT16
3	55504013	55504634	-	ENSG00000114251	WNT5A
3	55507986	55508964	-	ENSG00000114251	WNT5A
3	55516480	55517392	-	ENSG00000114251	WNT5A
3	55518269	55518844	-	ENSG00000114251	WNT5A
3	55520094	55522160	-	ENSG00000114251	WNT5A
3	55522369	55523836	-	ENSG00000114251	WNT5A

Table S3. AP-1 consensus motifs identified in FRA1^{EMT} genes.

Sequence name	Start	Stop	Score	P-value	Matched sequence
CD109	599	590	16.7038	9.09E-07	ATGAGTCACC
EPCAM	926	917	16.7038	9.09E-07	ATGAGTCACC
LEF1	764	773	16.7038	9.09E-07	ATGAGTCACC
FZD3	433	442	16.7038	9.09E-07	ATGAGTCACC
CLDN4	886	895	15.5695	2.56E-06	CTGAGTCACC
BMP7	894	885	15.5695	2.56E-06	CTGAGTCACC
SMAD3	195	204	15.5695	2.56E-06	CTGAGTCACC
ITGA3	135	144	15.5695	2.56E-06	CTGAGTCACC
RAC2	1373	1382	15.5695	2.56E-06	CTGAGTCACC
SERPINE1	437	428	15.5695	2.56E-06	CTGAGTCACC
CLDN4	815	824	15.4277	4.42E-06	GTGAGTCACC
BMP4	572	581	15.4277	4.42E-06	GTGAGTCACC
BMP7	840	831	15.4277	4.42E-06	GTGAGTCACC
AXL	647	638	15.4277	4.42E-06	GTGAGTCACC
TJP2	462	453	15.4277	4.42E-06	GTGAGTCACC
STAT3	679	670	15.4277	4.42E-06	GTGAGTCACC
VIM	511	520	15.4277	4.42E-06	GTGAGTCACC
PDGFB	838	829	15.4277	4.42E-06	GTGAGTCACC
CLDN4	970	961	15.1441	6.07E-06	ATGAGTCAGC
AXL	217	208	15.1441	6.07E-06	CTGACTCACC
AXL	440	449	15.1441	6.07E-06	ATGAGTCAGC
ZEB1	495	486	15.1441	6.07E-06	CTGACTCACC
WNT10B	192	201	15.1441	6.07E-06	CTGACTCACC
BMP4	580	571	15.0023	7.92E-06	GTGACTCACC
BMP7	2508	2517	15.0023	7.92E-06	GTGACTCACC
LAMC2	440	431	15.0023	7.92E-06	GTGACTCACC
PLAUR	50	59	15.0023	7.92E-06	GTGACTCACC
VIM	519	510	15.0023	7.92E-06	GTGACTCACC
TJP3	310	319	15.0023	7.92E-06	GTGACTCACC
TNC	619	610	14.7188	9.94E-06	ATGACTCAGC
EGFR	258	249	14.4352	1.22E-05	ATGACTCACA
PDGFB	1152	1143	14.4352	1.22E-05	ATGAGTCACT
LAMB3	1329	1320	14.0098	1.58E-05	CTGAGTCAGC
STAT5B	1496	1505	14.0098	1.58E-05	ATGACTCACT
ITGAV	70	79	14.0098	1.58E-05	ATGACTCACT

MMP14	48	57	14.0098	1.58E-05	CTGAGTCAGC
MMP16	273	282	14.0098	1.58E-05	CTGAGTCAGC
LAMB3	1247	1238	13.8681	1.75E-05	GTGAGTCAGC
ITGA3	1170	1179	13.8681	1.75E-05	CTGACTCATC
CDH1	876	867	13.8681	1.75E-05	GTGAGTCAGC
EPCAM	918	927	13.7263	1.93E-05	GTGACTCATC
RAC2	477	486	13.7263	1.93E-05	CTGAGTCACA
LAMC2	432	441	13.5845	2.23E-05	GTGAGTCACA
STAT5B	555	546	13.5845	2.23E-05	CTGACTCAGC
ITGA3	1071	1062	13.5845	2.23E-05	CTGACTCAGC
MMP14	56	47	13.5845	2.23E-05	CTGACTCAGC
TJP3	1150	1159	13.5845	2.23E-05	CTGACTCAGC
OCLN	241	250	13.5845	2.23E-05	CTGACTCAGC
PDGFB	818	827	13.5845	2.23E-05	GTGAGTCACA
PDGFB	194	203	13.5845	2.23E-05	CTGACTCAGC
SMAD3	203	194	13.4427	2.30E-05	GTGACTCAGC
ITGA3	143	134	13.4427	2.30E-05	GTGACTCAGC
CLDN4	107	98	13.3009	2.60E-05	CTGAGTCACT
BMP7	637	628	13.3009	2.60E-05	CTGAGTCACT
AXL	392	383	13.3009	2.60E-05	CTGAGTCACT
FBN1	91	100	13.3009	2.60E-05	ATGAGTCAGA
LAMB3	1239	1248	13.3009	2.60E-05	CTGACTCACA
ITGA3	1178	1169	13.3009	2.60E-05	ATGAGTCAGA
EGFR	81	90	13.3009	2.60E-05	CTGACTCACA
CLDN4	911	902	13.1591	3.05E-05	GTGAGTCACT
CLDN4	903	912	13.1591	3.05E-05	GTGACTCACA
BMP7	2516	2507	13.1591	3.05E-05	GTGAGTCACT
BMP7	832	841	13.1591	3.05E-05	GTGACTCACA
AXL	158	149	13.1591	3.05E-05	GTGACTCACA
TGFBR2	317	326	13.1591	3.05E-05	GTGAGTCACT
STAT5B	396	387	13.1591	3.05E-05	GTGACTCACA
STAT5B	388	397	13.1591	3.05E-05	GTGAGTCACT
STAT3	671	680	13.1591	3.05E-05	GTGACTCACA
ROBO1	723	732	13.1591	3.05E-05	GTGAGTCACT
ROBO1	418	427	13.1591	3.05E-05	GTGAGTCACT
TJP3	318	309	13.1591	3.05E-05	GTGAGTCACT
TJP3	1131	1122	13.1591	3.05E-05	GTGAGTCACT
TJP3	1123	1132	13.1591	3.05E-05	GTGACTCACA
PDGFB	826	817	13.1591	3.05E-05	GTGACTCACA

PDGFB	830	839	13.1591	3.05E-05	GTGACTCACA
CD109	335	326	12.8756	3.47E-05	CTGACTCACT
TGFBR2	29	38	12.8756	3.47E-05	ATGAGTCAGT
CDH1	868	877	12.8756	3.47E-05	CTGACTCACT
PDGFB	55	46	12.8756	3.47E-05	CTGACTCACT
CLDN4	823	814	12.7338	3.70E-05	GTGACTCACT
TGFBR2	325	316	12.7338	3.70E-05	GTGACTCACT
BMP4	414	423	12.592	3.79E-05	TTGAGTCACC
CDH1	35	26	12.4502	4.01E-05	ATGACTCAGT
TJP3	1256	1247	12.4502	4.01E-05	ATGACTCAGT
BMP7	572	563	12.1666	4.30E-05	CTGAGTCAGA
AXL	357	348	12.1666	4.30E-05	CTGAGTCAGA
LAMB3	1851	1860	12.1666	4.30E-05	CTGAGTCAGA
STAT5B	547	556	12.1666	4.30E-05	CTGAGTCAGA
PDGFB	1121	1112	12.1666	4.30E-05	CTGAGTCAGA
SERPINE1	163	172	12.1666	4.30E-05	CTGAGTCAGA
LAMB3	116	125	12.0249	4.70E-05	ATGAGTCACG
SNAI2	356	365	12.0249	4.70E-05	GTGAGTCAGA
CDH1	27	36	12.0249	4.70E-05	CTGAGTCATT
PDGFB	47	56	12.0249	4.70E-05	GTGAGTCAGA
EGFR	250	259	11.8831	4.92E-05	GTGAGTCATT
BMP7	564	573	11.7413	5.20E-05	CTGACTCAGA
SMAD3	914	923	11.7413	5.20E-05	CTGACTCAGA
NRP1	255	264	11.7413	5.20E-05	CTGAATCACC
PDGFB	202	193	11.7413	5.20E-05	CTGAGTCAGT
CLDN4	414	405	11.5995	5.81E-05	GTGAATCACC
CLDN4	894	885	11.5995	5.81E-05	GTGACTCAGA
CD109	610	601	11.5995	5.81E-05	ATGAATCATC
BMP7	886	895	11.5995	5.81E-05	GTGACTCAGA
TGFBR2	37	28	11.5995	5.81E-05	CTGACTCATT
ETS1	240	249	11.5995	5.81E-05	GTGAATCACC
STAT5B	222	231	11.5995	5.81E-05	GTGAATCACC
LAMB1	312	321	11.5995	5.81E-05	GTGAATCACC
TJP2	497	506	11.5995	5.81E-05	GTGAATCACC
ZEB1	487	496	11.5995	5.81E-05	GTGAGTCAGT
RAC2	485	476	11.5995	5.81E-05	GTGACTCAGA
RAC2	1381	1372	11.5995	5.81E-05	GTGACTCAGA
TJP3	1611	1602	11.5995	5.81E-05	GTGAATCACC
EGFR	89	80	11.5995	5.81E-05	GTGAGTCAGT

SERPINE1	429	438	11.5995	5.81E-05	GTGACTCAGA
LAMB3	124	115	11.4577	5.92E-05	GTGACTCATT
CD109	122	113	11.3159	6.23E-05	ATGAATCAGC
LAMC2	481	472	11.3159	6.23E-05	CTGACTCAGT
LAMB3	1859	1850	11.3159	6.23E-05	CTGACTCAGT
LAMB3	1321	1330	11.3159	6.23E-05	CTGACTCAGT
PLAUR	625	634	11.3159	6.23E-05	CTGACTCAGT
STAT5B	144	135	11.3159	6.23E-05	CTGACTCAGT
PDGFB	744	753	11.3159	6.23E-05	CTGACTCAGT
SERPINE1	171	162	11.3159	6.23E-05	CTGACTCAGT
AXL	277	268	11.0324	6.66E-05	ATGAATCACA
ROBO1	386	377	11.0324	6.66E-05	ATGAATCACA
AXL	150	159	10.7488	7.36E-05	GTGAGTCACG
LAMB3	162	153	10.7488	7.36E-05	ATTACTCACC
PLAUR	58	49	10.7488	7.36E-05	GTGAGTCACG
PDGFB	282	291	10.7488	7.36E-05	ATGAGTCATG
PDGFB	40	49	10.7488	7.36E-05	GTGAGTCACG
ROBO1	72	63	10.607	7.58E-05	ATGAATCACT
ROBO1	444	453	10.607	7.58E-05	ATGAATCACT
CLDN4	342	351	10.4652	7.97E-05	CTGACTCACG
PLAUR	1854	1845	10.4652	7.97E-05	ATGAGTCAGG
ITGA5	973	982	10.4652	7.97E-05	ATGACTCAAC
SNAI2	364	355	10.4652	7.97E-05	CTGACTCACG
AXL	639	648	10.3234	8.49E-05	GTGACTCACG
TJP2	454	463	10.3234	8.49E-05	GTGACTCACG
TJP2	341	332	10.3234	8.49E-05	GTGAATCATC
ROBO1	731	722	10.3234	8.49E-05	GTGACTCACG
ROBO1	426	417	10.3234	8.49E-05	GTGACTCACG
ITGB3	461	452	10.3234	8.49E-05	ATGACTCATG
PDGFB	290	281	10.3234	8.49E-05	ATGACTCATG
PDGFB	48	39	10.3234	8.49E-05	GTGACTCACG
VIM	533	524	10.0399	8.85E-05	CTTAGTCACC
OCLN	1071	1080	10.0399	8.85E-05	CTTAGTCACC
CLDN4	26	17	9.89808	9.30E-05	CTGAATCACA
PLAU	658	667	9.89808	9.30E-05	TTGACTCACT
ITGAV	414	423	9.89808	9.30E-05	CTGAATCACA
NRP1	226	235	9.89808	9.30E-05	TTGACTCACT
NRP1	360	351	9.89808	9.30E-05	TTGACTCACT
ITGBL1	138	147	9.89808	9.30E-05	TTGACTCACT

TGFBR2	4	13	9.7563	9.66E-05	ATGAATCATA
STAT3	57	66	9.7563	9.66E-05	ATGAATCATA
CDH4	3633	3642	14.7188	9.94E-06	ATGACTCAGC
CDH4	3641	3632	14.2934	1.31E-05	CTGAGTCATC
CDH4	2613	2622	14.1516	1.40E-05	GTGAGTCATC
CDH4	2621	2612	14.0098	1.58E-05	ATGACTCACT
CDH4	941	932	13.8681	1.75E-05	CTGACTCATC
CDH4	2527	2536	13.3009	2.60E-05	CTGAGTCACT
CDH4	933	942	13.3009	2.60E-05	ATGAGTCAGA
CDH4	27	18	12.7338	3.70E-05	ATGACTCATT
CDH4	393	402	12.4502	4.01E-05	ATGACTCAGT
CDH4	819	828	12.1666	4.30E-05	TTGACTCACC
CDH4	19	28	10.7488	7.36E-05	ATGAGTCATG
CDH4	3693	3684	10.4652	7.97E-05	CTGAATCATC
CDH4	441	450	9.89808	9.30E-05	CTGAATCACA
CDH4	1501	1510	9.89808	9.30E-05	CTGAATCACA
CDH4	2879	2870	9.7563	9.66E-05	ATGAATCATA

Table S4. Mean gene expression levels (log2) for the two main groups resulting from unsupervised clustering of stage B and C colorectal cancers using FRA1^{EMT} genes. P-values are for t-tests and adjusted for multiple testing using the Benjamini and Hochberg multiple testing correction.

Gene name	Affymetrix probe ID	Mean log2 expression in the right branch	Mean log2 expression in the left branch	P-value	Right vs left branch	shFRA1 vs shNS
VIM	1555938_x_at	4.8	5.3	3.26X10^06	down	down
VIM	201426_s_at	11.4	12.4	1.08X10^25	down	down
TGFBI	201506_at	12.0	12.2	1.89X10^01	down	down
FN1	1558199_at	5.6	6.1	2.31X10^14	down	down
FN1	210495_x_at	11.5	12.9	1.26X10^24	down	down
FN1	211719_x_at	11.6	12.9	2.64X10^23	down	down
FN1	212464_s_at	11.2	12.6	8.71X10^25	down	down
FN1	214701_s_at	7.6	8.6	5.23X10^14	down	down
FN1	214702_at	5.0	5.5	2.38X10^11	down	down
FN1	216442_x_at	11.5	13.0	3.52X10^25	down	down
LPXN	216250_s_at	8.2	8.5	1.47X10^05	down	down
LPXN	242778_at	5.7	5.7	8.95X10^02	down	down
NRP1	1561365_at	4.0	4.1	1.16X10^03	down	down
NRP1	210510_s_at	6.6	6.9	6.53X10^06	down	down
NRP1	210615_at	5.2	5.2	4.17X10^01	down	down
NRP1	212298_at	7.4	8.5	2.14X10^19	down	down
SNAI2	213139_at	7.4	8.6	1.45X10^26	down	down
FBN1	202765_s_at	7.8	9.2	1.40X10^28	down	down
FBN1	202766_s_at	8.5	9.9	1.40X10^29	down	down
FBN1	235318_at	5.6	6.8	1.08X10^25	down	down
ROBO1	213194_at	8.2	8.8	8.23X10^06	down	down
TNC	201645_at	8.2	9.5	1.38X10^15	down	down
TNC	216005_at	5.1	5.8	2.40X10^10	down	down
LEF1	210948_s_at	5.7	6.0	8.79X10^03	down	down
LEF1	221557_s_at	5.9	5.8	3.25X10^03	up	down
LEF1	221558_s_at	8.1	8.5	7.52X10^03	down	down
LAMC2	202267_at	8.6	8.9	3.25X10^03	down	down
LAMC2	207517_at	6.3	6.5	7.94X10^04	down	down
TGFB2	209908_s_at	4.5	4.6	3.69X10^02	down	down
TGFB2	209909_s_at	4.4	4.6	2.63X10^02	down	down
TGFB2	220406_at	6.6	6.6	6.96X10^01	down	down
TGFB2	220407_s_at	6.4	6.5	1.89X10^02	down	down
TGFB2	228121_at	6.9	7.5	4.45X10^11	down	down
SERPINE1	1568765_at	5.8	6.1	1.72X10^07	down	down
SERPINE1	202627_s_at	7.6	8.7	2.64X10^21	down	down

SERPINE1	202628_s_at	7.4	8.6	4.68X10^20	down	down
AXL	202685_s_at	7.7	7.8	3.48X10^02	down	down
AXL	202686_s_at	7.7	8.5	3.47X10^16	down	down
MMP14	160020_at	8.7	9.1	1.66X10^10	down	down
MMP14	202827_s_at	7.9	8.3	1.30X10^06	down	down
MMP14	202828_s_at	8.0	8.4	9.90X10^09	down	down
MMP14	217279_x_at	8.0	8.2	2.20X10^09	down	down
PLAU	205479_s_at	8.5	9.4	3.32X10^16	down	down
PLAU	211668_s_at	8.0	8.8	3.47X10^16	down	down
TGFBR2	207334_s_at	6.9	7.1	1.00X10^02	down	down
TGFBR2	208944_at	9.9	10.1	1.76X10^02	down	down
LAMB3	209270_at	9.0	9.0	4.71X10^01	down	down
JUN	201464_x_at	11.3	11.5	1.25X10^02	down	down
JUN	201465_s_at	8.7	8.3	4.71X10^03	up	down
JUN	201466_s_at	10.1	10.0	4.23X10^01	up	down
ITGA2	205032_at	8.9	8.9	8.24X10^01	up	down
ITGA2	227314_at	10.0	9.9	5.58X10^01	up	down
LAMA4	202202_s_at	8.4	9.2	8.00X10^22	down	down
LAMA4	210089_s_at	6.7	7.1	1.11X10^10	down	down
LAMA4	210989_at	4.6	4.7	1.00X10^02	down	down
LAMA4	210990_s_at	6.3	6.5	3.56X10^07	down	down
LAMA4	216081_at	6.6	6.4	1.03X10^02	up	down
ZEB1	210875_s_at	5.9	7.0	2.19X10^19	down	down
ZEB1	212758_s_at	7.2	7.6	8.62X10^08	down	down
ZEB1	212764_at	8.0	9.1	4.77X10^18	down	down
ZEB1	239952_at	6.1	6.3	3.02X10^07	down	down
PLAUR	210845_s_at	9.5	10.0	1.71X10^08	down	down
PLAUR	211924_s_at	8.9	9.4	1.10X10^07	down	down
PLAUR	214866_at	7.9	8.1	2.81X10^02	down	down
MMP16	207012_at	4.6	4.8	2.69X10^05	down	down
MMP16	207013_s_at	7.0	7.1	2.03X10^02	down	down
MMP16	208166_at	4.2	4.3	4.49X10^01	down	down
MMP16	208167_s_at	5.0	5.0	1.89X10^01	down	down
MMP16	223614_at	5.3	5.8	8.95X10^12	down	down
ITGB3	204625_s_at	5.5	5.4	1.54X10^02	up	down
ITGB3	204626_s_at	6.5	6.6	2.88X10^01	down	down
ITGB3	204627_s_at	5.4	5.8	8.75X10^14	down	down
ITGB3	204628_s_at	7.1	7.0	6.21X10^02	up	down
ITGB3	211579_at	5.5	5.5	9.06X10^01	up	down
ITGB3	215240_at	3.8	3.8	1.22X10^01	down	down
ITGB3	216261_at	6.4	6.5	7.94X10^04	down	down
ETS1	1555355_a_at	7.1	7.7	4.62X10^10	down	down
ETS1	214447_at	7.2	7.4	1.98X10^03	down	down
ETS1	224833_at	9.5	10.1	2.03X10^18	down	down
STAT5B	1555086_at	8.2	8.2	2.11X10^01	down	down
STAT5B	1555088_x_at	8.4	8.4	2.27X10^01	down	down
STAT5B	205026_at	6.5	6.6	2.59X10^01	down	down

STAT5B	212549_at	8.3	8.5	8.38X10^05	down	down
STAT5B	212550_at	7.5	7.5	6.61X10^01	down	down
ITGBL1	1557079_at	3.6	3.7	1.45X10^03	down	down
ITGBL1	1557080_s_at	5.3	6.2	4.42X10^08	down	down
ITGBL1	205422_s_at	5.8	7.1	2.77X10^12	down	down
ITGBL1	214927_at	6.4	7.1	1.12X10^08	down	down
ITGBL1	231993_at	4.9	5.6	2.85X10^08	down	down
LAMB1	201505_at	9.3	9.5	2.74X10^03	down	down
LAMB1	211651_s_at	9.0	9.3	2.45X10^03	down	down
ITGA5	201389_at	8.1	8.7	2.34X10^16	down	down
RAC2	207419_s_at	7.5	7.7	3.26X10^02	down	down
RAC2	213603_s_at	9.2	9.5	2.18X10^04	down	down
ITGA3	201474_s_at	8.1	8.1	8.26X10^01	down	down
LAMC1	200770_s_at	9.1	9.4	1.72X10^07	down	down
LAMC1	200771_at	9.8	10.4	1.85X10^19	down	down
WNT5A	205990_s_at	9.1	9.5	4.71X10^03	down	down
WNT5A	213425_at	8.1	8.4	1.46X10^02	down	down
WNT5A	231227_at	6.4	6.6	6.76X10^02	down	down
SMAD3	205396_at	6.7	6.8	1.54X10^01	down	down
SMAD3	205397_x_at	7.3	7.3	6.61X10^01	up	down
SMAD3	205398_s_at	7.3	7.4	1.54X10^02	down	down
SMAD3	218284_at	8.6	8.7	4.64X10^01	down	down
RND3	212724_at	10.5	10.6	4.98X10^02	down	down
TJP2	202085_at	10.5	10.4	3.06X10^02	up	down
TJP2	232017_at	7.8	7.8	8.43X10^01	up	down
TJP2	237132_at	5.3	5.3	7.75X10^01	down	down
TJP2	242991_at	5.1	5.1	1.62X10^02	up	down
TPM1	1558532_at	5.7	5.8	2.15X10^01	down	down
TPM1	206116_s_at	10.0	10.6	1.22X10^10	down	down
TPM1	206117_at	6.8	7.3	2.27X10^11	down	down
TPM1	210986_s_at	11.7	12.1	2.69X10^10	down	down
TPM1	210987_x_at	11.0	11.5	3.54X10^14	down	down
TPM1	238688_at	8.0	8.1	3.42X10^02	down	down
CDH4	1563587_at	5.9	5.8	3.11X10^04	up	down
CDH4	206866_at	5.7	5.5	8.42X10^04	up	down
CDH4	220227_at	6.4	6.3	9.55X10^02	up	down
CDH4	239485_at	6.2	6.1	6.10X10^02	up	down
ITGAV	202351_at	10.7	11.2	5.67X10^14	down	down
ITGA6	201656_at	10.9	10.8	1.24X10^01	up	down
ITGA6	215177_s_at	10.3	10.2	4.64X10^01	up	down
PCDHB9	223927_at	7.1	7.2	1.76X10^02	down	down
PCDH18	225975_at	8.7	9.1	2.01X10^04	down	down
PCDH18	225977_at	7.1	7.2	1.03X10^01	down	down
STAT3	208991_at	11.1	11.1	2.25X10^01	down	down
STAT3	208992_s_at	10.2	10.3	2.27X10^01	down	down
STAT3	225289_at	9.3	9.4	1.22X10^01	down	down
STAT3	243213_at	6.8	6.9	4.01X10^03	down	down

EGFR	1565483_at	9.5	9.9	2.17X10^02	down	down
EGFR	1565484_x_at	9.2	9.7	1.34X10^02	down	down
EGFR	201983_s_at	9.0	8.6	7.06X10^05	up	down
EGFR	201984_s_at	8.5	8.3	3.54X10^02	up	down
EGFR	210984_x_at	8.2	7.9	7.00X10^08	up	down
EGFR	211550_at	6.2	6.1	2.45X10^02	up	down
EGFR	211551_at	6.5	6.4	4.42X10^02	up	down
EGFR	211607_x_at	7.8	7.6	3.13X10^06	up	down
LAMA3	1560078_at	6.3	6.4	6.76X10^02	down	down
LAMA3	1563772_a_at	6.5	6.4	6.39X10^03	up	down
LAMA3	1568879_a_at	7.2	7.2	3.88X10^01	down	down
LAMA3	203726_s_at	8.8	9.0	2.87X10^01	down	down
LAMA3	234608_at	5.8	5.9	2.75X10^01	down	down
LAMA3	234719_at	5.9	5.8	3.57X10^03	up	down
OCLN	209925_at	8.1	7.9	1.11X10^01	up	up
OCLN	227492_at	10.3	10.0	1.06X10^04	up	up
OCLN	231022_at	8.5	8.2	2.69X10^05	up	up
FZD3	219683_at	6.9	7.0	4.03X10^01	down	up
FZD3	227499_at	5.7	5.7	6.42X10^01	up	up
FZD3	227524_at	7.5	7.3	7.94X10^04	up	up
FZD3	239082_at	8.6	8.6	7.75X10^01	down	up
TJP3	213412_at	7.8	7.4	3.35X10^09	up	up
TJP3	35148_at	8.3	8.0	6.47X10^07	up	up
PDGFB	204200_s_at	6.5	6.7	2.19X10^08	down	up
PDGFB	216055_at	5.4	5.4	7.61X10^01	down	up
PDGFB	216061_x_at	7.4	7.5	9.96X10^04	down	up
PDGFB	217112_at	5.7	5.5	3.35X10^07	up	up
RAC3	206103_at	6.2	6.2	8.82X10^01	up	up
BMP7	209590_at	7.4	7.4	8.19X10^01	up	up
BMP7	209591_s_at	6.4	6.4	9.64X10^01	down	up
BMP7	211259_s_at	6.2	6.0	3.43X10^02	up	up
BMP7	211260_at	7.1	6.8	5.16X10^08	up	up
BMP4	211518_s_at	9.4	9.3	6.69X10^01	up	up
TPM2	204083_s_at	9.3	10.4	2.03X10^18	down	up
TPM2	212654_at	6.4	6.7	8.08X10^06	down	up
TPM2	227397_at	6.4	6.3	5.82X10^03	up	up
TPM2	229911_at	6.4	6.3	5.62X10^03	up	up
CLDN4	1569421_at	7.2	7.2	4.93X10^01	down	up
CLDN4	201428_at	10.2	10.0	9.87X10^03	up	up
WNT16	221113_s_at	6.6	6.6	3.81X10^01	down	up
WNT16	224022_x_at	3.7	3.8	1.11X10^01	down	up
CDH1	201130_s_at	9.0	8.8	1.20X10^01	up	up
CDH1	201131_s_at	12.0	11.7	1.98X10^03	up	up
CLDN7	202790_at	11.0	10.8	1.34X10^02	up	up
RAB25	218186_at	10.6	10.4	8.53X10^04	up	up
CD109	226545_at	6.8	8.1	6.95X10^14	down	up
CD109	229900_at	6.0	6.4	9.69X10^08	down	up

CD109	239719_at	6.7	6.7	3.82X10^01	down	up
EPCAM	201839_s_at	12.9	12.7	6.38X10^03	up	up

Table S5. Clinicopathological and molecular associations for *FOSL1* expression levels in stage B and C CRC patients. P-values are for the $\chi 2$ test for categorical variables and the Wilcoxon rank-sum test for continuous variables.

Characteristic	All cases N	*FOSL1*^{low} N (%)	*FOSL1*^{high} N (%)	P-value
Total	185	146 (80.2)	36 (19.8)	
FRA1^{EMT} signature				
Epithelial	149	81 (85.3)	14 (14.7)	
Mesenchymal	36	68 (74.7)	22(25.3)	0.11
Age				
Median (range)	67, 26-92	67 (26-92)	69 (30-90)	0.679
Gender				
Male	98	83 (84.7)	15 (15.3)	0.169
Female	87	66 (75.9)	21 (24.1)	
Tumour location				
Colon	163	129 (79.1)	34 (20.9)	0.307
Rectum	22	20 (90.9)	2 (9.1)	
Tumor stage				
B	91	70 (76.9)	21 (23.1)	0.3
C	94	79 (84.0)	15 (16.0)	
T stage				
2	9	9 (100)	0 (0)	
3	163	133 (81.6)	30 (18.4)	
4	13	7 (53.8)	6 (46.2)	**0.017***
N stage				
0	91	76 (83.5)	15 (16.5)	
1	66	51 (77.3)	15 (22.7)	
2	25	19 (76.0)	6 (24.0)	0.531
Unknown	3	-	-	

Table S6. Clinicopathological and molecular associations for FRA1^{EMT} signature in stage B and C CRC patients. P-values are for the $\chi 2$ test for categorical variables and the Wilcoxon rank-sum test for continuous variables.

Characteristic	All cases N	Epithelial signature N (%)	Mesenchymal signature N (%)	P-value
Total	185	95 (51.3)	90 (48.6)	
FRA1^{EMT} signature				
Epithelial	149	81 (54.4)	68 (45.6)	
Mesenchymal	36	14 (38.9)	22 (61.1)	0.139
Age				
Median (range)	67 (26-92)	70 (26-92)	64 (26-92)	0.005*
Gender				
Male	98	82 (54.1)	45 (45.9)	0.521
Female	87	42 (48.3)	21 (51.7)	
Tumour location				
Colon	163	82 (50.3)	81 (49.7)	0.585
Rectum	22	13 (59.1)	9 (40.9)	
Tumor stage				
B	94	52 (55.3)	42 (44.7)	0.342
C	91	43 (47.3)	48 (52.7)	
T stage				
2	9	6 (66.7)	3 (33.3)	0.622
3	163	82 (50.3)	81 (49.7)	
4	13	7 (53.8)	6 (46.2)	
N stage				
0	91	52 (57.1)	39 (42.9)	
1	66	37 (56.1)	29 (43.9)	
2	25	6 (24.0)	19 (76.0)	**0.0098***
Unknown	3	-	-	

Table S7. Univariate and multivariate Cox proportional-hazards analysis of survival for stage B and C colorectal cancer patients according to *FOSL1* expression and the concordant FRA1^{EMT} gene expression patterns. P-values are for the Wald test.

			Univariate model		Multivariate model	
Molecular Feature	N	Events/ years obs.	HR (95% CI)	P-value	HR (95% CI)	P-value
Total	182	47/640.2				
Classifier						
FOSL1^{low}/epithelial	81	10/300.0	1 (Referent)		1 (Referent)	
FOSL1^{high}/epithelial	14	5/45.95	3.26 (1.11-9.53)	0.03	3.38 (1.10-10.46)	0.03
FOSL1^{low}/mesenchymal	65	14/44.22	2.12 (0.97-4.64)	0.06	1.70 (0.75-1.17)	0.21
FOSL1^{high}/mesenchymal	22	18/250.1	8.16 (3.60-18.49)	<0.001	7.06 (3.03-16.48)	<0.01
Age						
Age in decades			0.90 (0.73-1.11)	0.322	0.94 (0.75-1.17)	0.56
Gender						
Male	97	27/347.7	1 (Referent)		1 (Referent)	
Female	85	20/292.5	0.87 (0.49-1.55)	0.635	0.91 (0.48-1.71)	0.76
Tumor location						
Colon	160	41/556.1	1 (Referent)		1 (Referent)	
Rectum	22	6/84.0	1.01 (0.43-2.39)	0.975	1.32 (0.52-3.33)	0.55
Tumor stage						
B	91	13/336.4	1 (Referent)		1 (Referent)	
C, N1	66	20/227.2	2.37 (1.18-4.76)	0.016	2.32 (1.07-5.05)	0.03*
C, N2	25	14/76.5	4.56 (2.14-9.71)	<0.001	3.06 (1.76-9.37)	0.001*
Adjuvant treatment						
No	98	20/269.8	1 (Referent)		1 (Referent)	
Yes	84	27/298.2	1.5 (0.84-2.68)	0.17	0.84 (0.42-1.67)	0.62

Table S8. List of qRT-PCR and ChIP primers used in this study.

Primers used for ChIP

Gene	Forward primer	Reverse primer	Product size
AXL	AGGAGGCAGGGGTGCTGAGA	ACGCCATGGGTGCCAAACTT	193bp
BMP4	TCTGCCTGTCTCCCCTCACC	TGGGATTCCCGTCCAAGCTA	164bp
BMP7	GGCCCTGGGTACTGAAGACG	GAGATGCAGCGCGAGATCCT	190bp
FN1	TGGGGCTGAACCATTTGCTG	TTTTCCCGCAGGTTGCGAAG	200bp
FOSL1	GCCTTCGACGTACCCCTGGA	TCCACCTGGTGCCAAGCATC	183bp
LEF1	TGGCCGGGATGATTTCAGAC	CCACAGCGGAGCGGAGATTA	232bp
SNAI2	TAATCCAGCCCAGCCCCATC	CGTCTGACTCACGCCATCAGC	223bp
TGFB2	TGGAGTAACCAAGCGGGTCA	GCGTGCGTGTGTGTGTGTGT	219bp
VIM	CCGCTAGGAGCCCTCAATCGG	TTCGGACGGCGGGAGTTGCG	126bp
ZEB1	CCTGGTCTCTCTCCGCCTAGC	AGAAGCAACAGCCGCTCCAC	151bp

Primers used for RT-PCR

Gene	Forward primer	Reverse primer	Product size
AXL	GAGAACATTAGTGCTACGCGGAA	CCTTAGCCCTATGTCCATTAGCA	124bp
BMP4	GCATTCGGTTACCAGGAATC	TGAGCCTTTCCAGCAAGTTT	106bp
BMP7	CTTCCCCTTCTGGGATCTTG	GATAGCCATTTCCTCACCGA	136bp
CDLN7	CACCAGGGAGACCACCATTA	ATGTACAAGGGGCTGTGGAT	132bp
EPCAM	AGAACCTACTGGATCATCATTGAA CTAA	CGCGTTGTGATCTCCTTCTG	100bp
FN1	CCATCAAGCCAGATGTCAGAAGCT	GGATGGTGCATCAATGGCA	139bp
FOSL1	CACCGACCCATCTGCAAA	CACTGGTACTGCCTGTGT	60bp
LAMC2	CAGCGGAATCCATTACCAGT	TGCTTCTCGCTCCTCCTG	128bp
SNAI2	CATGAGGAATCTGGCTGCTGTGTA	GCACAGGAGAAAATGCCTTTGGAC	123bp
TGFB2	CTCCATTGCTGAGACGTCAA	CGACGAAGAGTACTACGCCA	133bp
TGFBI	TTGAGAGTGGTAGGGCTGCT	CCAAAGGAAAATCTGTGGCA	107bp
VIM	CGCCAGATGCGTGAAATGG	ACCAGAGGGAGTGAATCCAGA	278bp
ZEB1	GCACCTGAAGAGGACCAGAG	TGGTGATGCTGAAAGAGACG	537bp
ZEB2	CAAGAGGCGCAAACAAGC	GGTTGGCAATACCGTCATCC	127bp
